# Heat Exposure and Multiple Sclerosis—A Regional and Temporal Analysis

**DOI:** 10.3390/ijerph18115962

**Published:** 2021-06-02

**Authors:** Gill Chacko, Sneh Patel, Anat Galor, Naresh Kumar

**Affiliations:** 1 Department of Public Health Sciences, University of Miami Miller School of Medicine, Miami, FL 33136, USA; gxc614@miami.edu; 2 Ophthalmology, Miami Veterans Affairs Medical Center, Miami, FL 33136, USA; sbpatel@med.miami.edu (S.P.); agalor@med.miami.edu (A.G.); 3 Bascom Palmer Eye Institute, University of Miami Miller School of Medicine, Miami, FL 33136, USA; 4 Research Services, Miami Veterans Affairs Medical Center, Miami, FL 33136, USA

**Keywords:** heat waves, multiple sclerosis, climate, extreme weather, heat stress

## Abstract

Multiple sclerosis (MS) is a neurological disorder that progressively distorts the myelination of axons within the central nervous system (CNS). Increased core body temperature or metabolism as a result of exercise are common causes of short-term exacerbations of neurological symptoms in MS. About 60–80% of patients with MS experience a worsening of their symptoms when exposed to heat. In comparison, less data are available on the relationship between ambient meteorological conditions (e.g., temperature and relative humidity (RH)) and fluctuations in such variables in relation to MS symptoms. Thus, this study examined associations between time-lagged exposure to meteorological conditions and risk of a clinic visit due to MS among US veterans between 2010 and 2013. This study leveraged data from the Veterans Affairs (VA) and National Climactic Data Center (NCDC) for the continental US, partitioned into eight climate zones. We used a case crossover design to assess the risk of a MS clinic visit with respect to several meteorological conditions. Location-specific time-lagged daily (ambient) exposure to temperature, RH, and temperature variations (standard deviation (SD) of temperature) were computed (up to 30 days) for each case (i.e., day of MS visit) and control (a randomly assigned date ± 90–270 days prior to visit). Statistical analyses were conducted to examine independent associations between the selected meteorological conditions and risk of MS visits at the national and regional levels. A total of 533,066 patient visits received a MS diagnosis (International Classifications of Diseases (ICD)-9 code = 340). The Northeast (NE) and Upper Midwest (UMW) regions reported the highest frequency of clinic visits due to MS. Clinic visits were 9% more likely to occur in the spring, summer, and fall months (March–October) than in the winter (OR = 1.089; 95% CI = 1.076–1.103; *p* < 0.01). In the univariate analyses, the SD of temperature, temperature, and temperature–RH interaction were positively associated with an elevated risk of a MS clinic visit, while the RH was negatively associated with the risk for a clinic visit. In multivariate analyses, the strongest association of a MS clinic visit was observed with the SD of the temperature (OR = 1.012; 95% CI 1.008–1.017; *p* < 0.01). These associations between MS clinic visits and meteorological conditions varied across climate regions, with the strongest associations being observed in the LMW, UMW, DSW, and NE zones. The SD of the temperature was again the strongest associated predictor when examined regionally. Temperature variations and temperature–RH interactions (a proxy of the heat index) showed significant associations with MS clinic visits. These associations varied across climate regions when examined geographically. Our findings have implications for the management of MS in severe or recurrent cases, especially considering the impending changes in the daily temperature variations and intensity of the heatwaves expected with the intensification of global warming.

## 1. Introduction

Multiple sclerosis (MS) is a neurological disorder characterized by disturbed axon myelination within the central nervous system (CNS) leading to altered, slowed, or blocked conduction pathways of CNS axons [1]. MS is typically diagnosed during the young adulthood years (mean age of diagnosis of 35 years), and is considered to be the most common disabling disease of young adults without an underlying traumatic etiology [2]. While the pathogenesis of the disease is not entirely understood, MS is believed to stem in part from a T-cell mediated autoimmune process—inflammatory infiltrates dominated by MHC class 1 CD8+ T-cells seen in tissue biopsy of individuals with MS are thought to directly lead to oligodendrocyte damage and demyelination as a result of local inflammation [2]. When immune responses are activated in individuals with MS, various changes are noted in the CNS including inflammation, demyelination, loss of axons and disarrangement [1]. Overall, these histopathologic changes can manifest as a myriad of paroxysmal symptoms (e.g., fatigue, reduced vision, double vision, muscle weakness, loss of sensation, spastic bladder) which can range from mild to severe [1].

Several factors have been implicated in the risk for the development of MS (e.g., incidence), including female gender, specific ethnicities, smoking, Epstein-Barr virus (EBV) infection, lower levels of vitamin D, and certain HLA gene haplotypes [3,4]. Interestingly, environmental factors have also been implicated in disease pathophysiology [1]. For example, a greater distance from the equator has been implicated in a higher MS prevalence compared to areas near the equator [5,6]. Supporting this idea, the prevalence of MS in Europe is over 100 per 100,000 persons, as compared to 10 per 100,000 persons in areas near the equator [5]. 

Literature has also examined how environmental factors relate to risk for MS symptoms (e.g., exacerbations), such as heat stress (e.g., increased core body temperature), with upwards of 60–80% of individuals with MS reporting worsening of symptoms when exposed to heat stress such as during physical activity [1,7]. Exacerbation of MS symptoms has been previously termed as Uhthoff’s phenomenon and can be described as transient worsening of neurological function in symptoms of multiple sclerosis (e.g., worsening visual acuity) in response to increases in core body temperature, hypothesized to be secondary to temperature-sensitive conduction blockade of partially demyelinated axons in MS lesions within the CNS [8]. While this connection is not entirely understood, hypotheses have been raised as to why heat stress may exacerbate MS [9]. Most notably, genetic variation in proteins related to adaptability to environmental factors may connect heat stress and MS. For example, heat shock proteins (HSP), proteins that act as chaperones (assisting in folding of newly synthesized proteins and degrading of unstable or misfolded proteins), are known to be upregulated during stress (e.g., sudden exposure to heatwave, hypoxia, and exposure to free-radicals or toxic metal ions) in order to maintain cellular homeostasis and survival [4,10]. Literature has consistently reported a relationship between increased synthesis of HSP70 and MS, [9,10]—as MS lesions trigger inflammation and oxidative stress in the CNS, the expression of several HSP proteins including HSP70 increases, and this increased release of HSP70 is thought to promote the T-cell immune response by either acting as an adjuvant for myelin peptides or as a proinflammatory cytokine [10,11]. This stress-induced wave of inflammation may explain the noted relationship between elevated body temperature and worsened MS symptoms. Less studied than HSP70, a null mutation in the gene encoding PLP1 (proteolipid protein 1), which normally functions to regulate thermal hyperalgesia, has also been associated with axonal degeneration and development of MS [12,13]. While the exact mechanism is less studied than that of HSP, animal models have shown that PLP1 mutations lead to apoptosis of oligodendrocytes (similar to T-lymphocyte mediated toxicity in MS) due to accumulation of PLP1 in the endoplasmic reticulum and the induction of the unfolded protein response (UPR), leading to demyelination and onset of MS-like symptoms [12,13].

Less established are the associations between MS and ambient environmental conditions, such as temperature, relative humidity (RH), and temperature variation. A handful of studies have examined these links, namely those regarding ambient temperature, with varying findings. It has been reported that up to 60–80% of MS patients experience worsening of symptoms (triggered short-term exacerbations of clinical signs symptoms of MS) when exposed to higher ambient temperature and/or increased core body temperature such as during exercise [1]. Supporting this statistic, a 5-year retrospective Irish study (1981–1985) of 87 patients with MS found that the duration of exacerbation was positively correlated with ambient temperature (r = 0.83, *p* < 0.001) [14]. Furthermore, an American study of 40 individuals with MS and 40 controls found that cognitive status (a combination score of processing speed [via Symbol Digit Modalities Test (SDMT)] and learning/memory [via Selective Reminding Test (SRT)]) in patients with MS was worsened when outdoor temperature increased (r = −0.45, *p* < 0.05) while this relationship was not seen in controls (r = 0.0, *p* = 0.98), with longitudinal cognitive decline over time (scores at baseline vs. after 6 months) as patients were exposed to prolonged increased ambient temperature (r = −0.39 *p* = 0.01) [15]. Results have not been entirely cohesive—a French study that examined MS hospital admissions over a 3 year period (2000–2003) which included a summer heatwave found that while a higher frequency of MS visits occurred during the summer compared to winter (OR = 1.04 vs. 0.85, *p* = 0.03), the associations between ambient temperature with MS admissions and MS exacerbations were insignificant (data not provided; *p* > 0.05) [16]. Similarly, a 14 year study (2001–2014) of 2000 patients in Iran found that both RH and ambient temperature in this area were not significantly related to risk of MS clinic visits (data not provided) [17].

Overall, while the associations between MS and environmental factors have been explored, findings have been variable across the literature. A more robust understanding of how exposure to temperature, RH, and temperature variation are warranted, especially considering that global warming is expected to lead to more erratic and extreme weather patterns, including increased frequency and intensity of heat waves [18]. In order to advance this literature, we leveraged national health and weather datasets to examine the associations between ambient meteorological conditions and MS clinic visit risk both across the continental US and within domestic climate regions. Our central hypothesis was that changes in ambient meteorological conditions, namely increase in ambient temperature, temperature variation, and simultaneous increase in temperature and RH, increased the risk of MS clinic visit due to exacerbation of the disease symptoms and signs. We further hypothesized that this risk varied across regions.

## 2. Materials and Methods

### 2.1. Study Population

This study included all veterans who visited any US Veteran Affair hospitals and/or clinics (VA) located in the continental US between January 2010 to December 2013. All veterans who were diagnosed with MS (by International Classification of Diseases, ICD-9 340) were included in this retrospective analysis. A total of 27,290 unique patients were seen during this period with most of them having multiple visits. During this period, a total of 530,075 visits received a MS ICD-9 code. Approval was obtained from the Miami VA Institution Review Board to allow the retrospective analysis of patient data (IRB protocol number: 3011.01). The study was conducted in accordance with the principles of the Declaration of Helsinki and complied with requirements of the US Health Insurance Portability and Accountability Act.

### 2.2. Method

We used a case-crossover design in which each visit (or encounter) to a VA clinic had a control on a randomly identified date between 90 to 270 days prior to the date of MS coded clinic visit. All data included the date and time of clinic visit, location of the clinic as well as patient’s demographics. All encounters were geocoded using latitude and longitude coordinates of each patient’s treatment facility. We used ArcGIS 10.8 [19] to display the distribution of MS clinic visits in the US. Since we did not have access to veteran population data for the catchment of each VA clinic/hospital, we computed MS visit encounters per 10,000 veterans visiting each clinic.

### 2.3. Meteorological Data

All meteorological data from 2009 to 2014 were acquired from the National Climactic Data Center (NCDC) [20]. Although the health data were from January 2010, we needed weather data for control dates randomly assigned on days within 3 and 9 months prior to the diagnosis dates. One of the main assumptions of our study is that all subjects seen in a VA clinic on a given day had the same ambient meteorological conditions around the clinic location on that day. Thus, we identified all meteorological stations within 0.75° distance (=82.5 km at equator) from the location of each clinic, and then computed daily averages and standard deviations (SD) of hourly measurements of the selected meteorological conditions for each day. Based on date and zip code both meteorological and clinical data were integrated, assigning daily meteorological data to all MS visits a VA clinic on a given day and corresponding controls days. Once the data were collocated, daily time-lagged exposures were computed for all (clinic) encounters who received multiple sclerosis diagnosis (*MS clinic visits* here to after) and controls for up to 30 days. In mathematical form, if *x_ijt_* is a meteorological condition, *x*, at/around a given zip code, *j*, on a given day, *t*, then time-lagged exposure for a given lag (*l*) is *x_ij(t-l)_*, i.e., the meteorological condition *l^th^* day before the diagnosis day for an encounter and the day randomly chosen as a control for a given MS visit.

### 2.4. Statistical Analysis

All analyses were conducted in STATA (StataCorp LLC, College Station, TX, USA) [21]. We conducted three sets of analyses: descriptive analyses of MS clinic visits by seasons and climate regions, multivariate analyses of the associations of meteorological conditions with MS clinic visits and variations in the associations of meteorological conditions (1) ambient temperature, (2) standard deviation (SD) of temperature [a proxy of temperature variation], (3) relative humidity (RH), and 4) temperature-RH interaction [a proxy of heat index] with MS clinic visits across climate regions in the US. We then stratified zip codes based on the eight climate regions: Desert Southwest (DSW), Lower Midwest (LMW), Northeast (NE), Pacific Northwest (PNW), Pacific Southwest (PSW), Southeast (SE), Subtropical (ST) and Upper Midwest (UMW). We conducted analyses for each climate region separately to assess region-specific association of time-lagged meteorological conditions with MS clinic visits. We used multivariate logistic regression adjusting for seasonality and weekday/weekend effects. We used cluster option for zip code and day assuming correlation in the occurrence of MS clinic visits within a zip code on a given day, and computed robust standard error (see logit function in STATA with cluster and robust options) [21]. We computed model parameters for each lag separately. Thus, we computed coefficients of each variable for each lag up to 30 days, which allowed us to examine changes in associations of meteorological conditions with the increase in time lag. For all models, statistical significance was considered at *p* ≤ 0.05, and likelihood of the occurrence of MS visit was reported as odds ratio (OR) with corresponding 95% confidence intervals (CI).

## 3. Results

### 3.1. Study Population & Clinic Visits

Between January 2010 and December 2013, a total of 27,290 patients made 530,075 visits and received an ICD-9 code for MS, all which were considered as cases for the purposes of our analyses. The average number of clinic visits per patient was 19.4, suggesting that MS patients required frequent access to medical care. The majority of the study population consisted of elderly, white, non-Hispanic males (mean age 59.5 years, 81.2% male, 92.0% non-Hispanic, 75.2% white, and 18.3% black).

**Figure 1:**
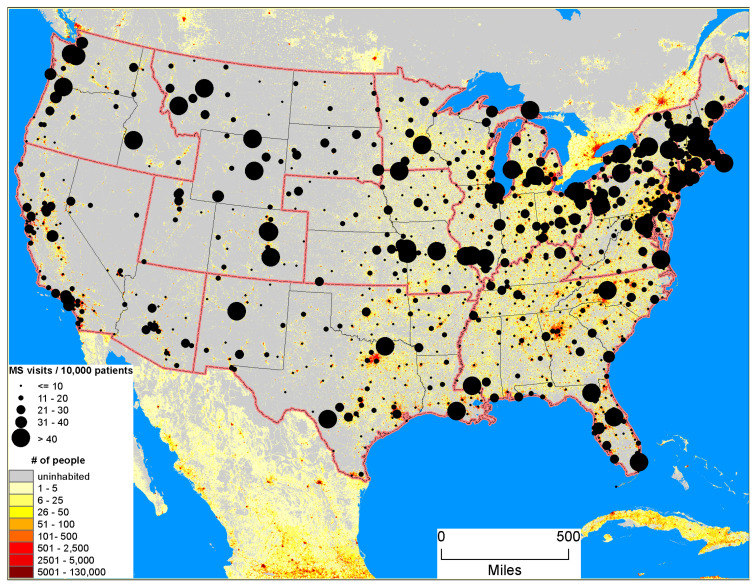
MS visits at VA clinics across the United States, 2010–2013.

Nationally, there were 39.4 clinic visits per 10,000 patient visits to VA clinics during the study period. However, visit rates varied across geographic regions. The Northeast and PNW zones, along with some areas of the Midwest regions, showed relatively higher rates of MS clinic visits (Figure 1; Table 1). The highest MS clinic visit rate of 67.6 per 10,000 patient visits was observed in PNW, followed by the NE (64/10,000) and ST (46.8/10,000 patients) zones. The lowest MS clinic visit rate was observed in the LMW (15.1 MS clinic visits/10,000 patients) followed by UMW (24.6 MS clinic visits/10,000 patients) zones.

The visit rates also varied across seasons (Table 2). Nationally, the highest frequency of MS clinic visits was observed in March (8.9%), followed by August (8.8%). In comparison, the winter months had a relatively low frequency of MS clinic visits as compared to other months, e.g., November (8.1%), December (7.7%), and January (8.0%). There were also regional variations in the frequency of MS clinic visits in the DSW, LMW, PNW, and SE zones. The highest frequency of MS clinic visits occurred in March (8.9%), followed by August (8.8%) (Table 2). Among all the regions, the desert SW reported the highest frequency of MS clinic visits (9.2%) in August. Overall, there are two seasonal peaks of MS clinic visits: spring (March and April) and early fall (August and September).

### 3.2. Seasonal and Regional Trends in Meteorological Conditions

The mean temperature in the US was 13.3 °C (SD 10.4 °C), and the mean RH was 68.0% (SD 16.3%). Stratifying these data by seasonality, the winter and fall months accounted for the highest standard deviations in the mean temperature (higher dispersion or fluctuations in the temperature), while the spring and summer months showed comparatively higher fluctuations in the RH (Table 3). These data were also examined by region—among all the climate zones, the ST region accounted for the least variation in temperature (mean 21.9 °C, SD 6.2 °C), followed by the PSW (mean 16.4 °C, SD 7.3 °C) and the PNW (mean 9.6 °C, SD 8.3 °C) (Table 3). On the other hand, the ST region accounted for the least variation in RH (mean 74.5%, SD 9.8%) (Table 4). However, this region had the highest average among all the regions. The UMW (mean 8.5 °C, SD 11.2 °C), LMW (mean 12.3 °C, SD 10.8 °C), and DSW (mean 16.0 °C, SD 10.4 °C) regions exhibited the highest variations in temperature, while the PSW (mean 54.1%, SD 23.2%) and PNW (mean 68.3%, SD 18.5%) zones showed the highest variation in RH. Although the PSW had the highest variation in RH, this region registered the lowest average RH among all the regions (Table 4).

### 3.3. Nationwide Associations

**Figure 2:**
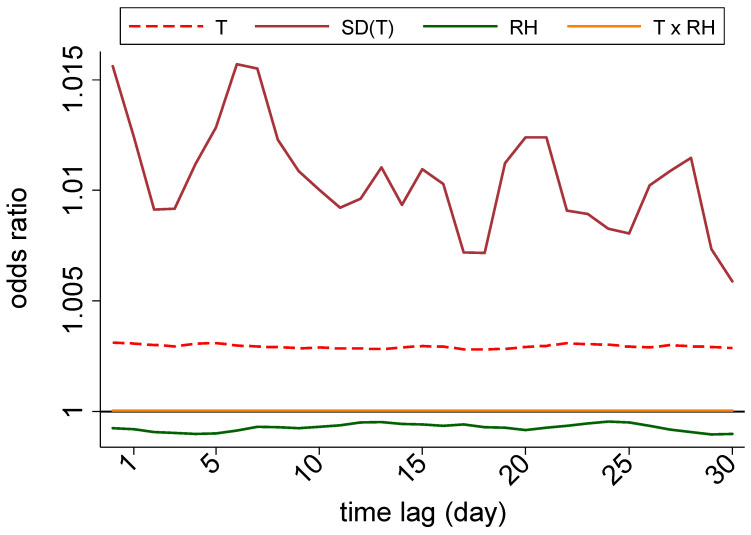
Daily time-lagged OR of MS visits with respect to the selected meteorological conditions. T = ambient temperature (°C); SD (T) = standard deviation of hourly ambient temperature (°C); RH = relative humidity (%).

In order to examine the nationwide associations between the MS clinic visit rates and each of the four variables (hourly ambient temperature, SD of hourly temperature, RH, and temperature–RH interactions), our analyses began with univariate analyses, with adjustments for weekend–weekday effects only (Figure 2 and Table S1 in the Supplementary Online Materials (SOM)). Across the US, independent exposure to all four variables showed significant associations with MS clinic visit risk at varying magnitudes and directions. Although there was a slight decline in the associations of all four meteorological conditions over a time lag, all four associations were significant for each of the 30-day lags. For the ambient temperature, a positive association was observed, with a 10 °C increase in temperature on a given day being associated with a 0.3% increased likelihood of a clinic visit (OR = 1.003; 95% CI 1.002–1.004; *p* < 0.01). The SD of the temperature represented the strongest association with MS clinic visits among all the meteorological conditions, with a one SD increase in the temperature on a given day being associated with as high as a 1.6% increased likelihood of a MS clinic visit on that day. However, the strength of this association changed with the increase in time lag, starting from its peak association on day lag 0 (OR = 1.016; 95% CI = 1.012–1.019; *p* < 0.01, each) and falling to its lowest value on day lag 30 (OR = 1.006; 95% CI = 1.002–1.010; *p* < 0.01). In comparison, a negative association with the RH was noted, with a 10% increase in RH on a given day being associated with a 0.08% lower likelihood of a MS clinic visit risk (OR = 0.9992; 95% CI = 0.99893–0.99952; *p* < 0.001). Finally, for the temperature–RH interaction effect (e.g., heat index), a simultaneous 10 °C increase in temperature and 10% increase in RH was associated with an increased risk, although to a much lesser degree than the other variables, with its strongest effect as OR = 1.00004; 95% CI = 1.000034–1.000047; *p* < 0.01. When stratifying these relationships by time of year, it was noted that the seasonality was also significantly associated with MS clinic visits—visits were 8.9% more likely to occur in the spring, summer, and fall months (March–October) in comparison to the winter (OR = 1.089; 95% CI 1.076–1.103; *p* < 0.01).

After the univariate analyses, we constructed a multivariate logistic regression model, which allowed for examination of the same associations while considering all variables concomitantly (Table 5). Like in the univariate analyses, the four associations were significant for all 30-day lags when examined nationally. First, the association between a MS clinic visit risk and the temperature varied in comparison to the univariate trends, namely in that it showed an inverse association with MS clinic visits in the multivariate analysis that adjusted for the RH and temperature–RH interactions. Specifically, this association reached its peak effect on day lag 1, with a 0.7% decreased likelihood for a visit (OR = 0.993, 95% CI 0.990–0.997, *p* < 0.01). Similarly, the SD of the temperature still showed a positive association with MS clinic visits, with a one SD increase in the hourly temperature being associated with a 1.2% higher likelihood of a MS clinic visit on day lag 0 (OR = 1.012; 95% CI 1.008–1.017; *p* < 0.01); this variable accounted for the strongest association with a clinic visit risk among all the variables. Next, on the other hand, the RH retained its inverse association, and it reached its peak effect on day lag 1 with a 0.2% decreased likelihood for a visit (OR = 0.998, 95% CI 0.997–0.999, *p* < 0.01). Finally, the temperature–RH interactions again showed a small positive association with MS clinic visits, with a simultaneous 10 °C increase in the temperature and 10% increase in the RH on being associated with an 0.01% higher likelihood for a clinic visit (OR = 1.000107; 95% CI 1.00006–1.000154; *p* < 0.01).

### 3.4. Region-Specific Associations

**Figure 3:**
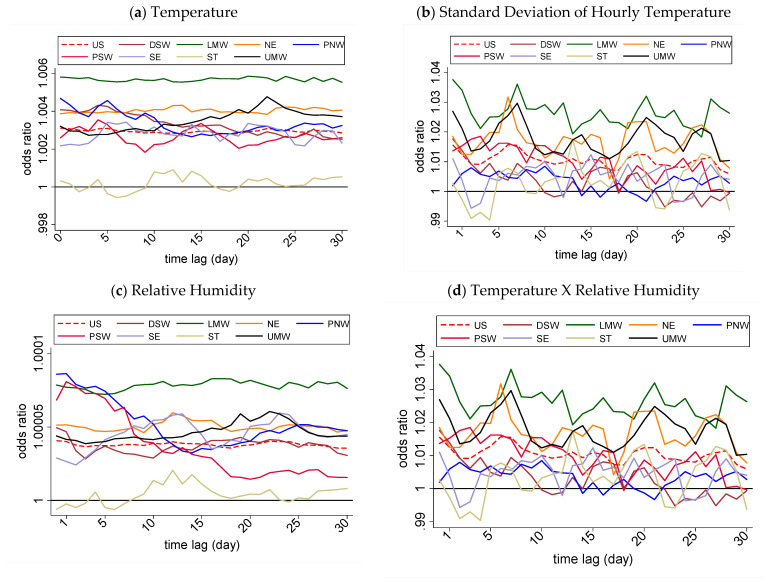
Associations between the time-lagged selected meteorological conditions and MS clinic visit by US veterans, 2010–2013.

The region-specific associations were also examined, first via univariate analyses (Figure 3 and Table S1 in the SOM). The time-lagged ambient temperature showed the strongest association with MS clinic visits in the LMW and was significant for all the 30-day lags (day lag 0: OR = 1.006; 95% CI = 1.004–1.008; *p* < 0.01), followed by the PNW (day lag 0: OR = 1.005; 95% CI = 1.000–1.009; *p* < 0.05), DSW (day lag 0: OR = 1.004; 95% = CI 1.001–1.007; *p* < 0.01), and NE (day lag 0: OR = 1.004; 95% CI = 1.002–1.006; *p* < 0.01) zones. In comparison, the same association in the UMW was significant for all 30 days (day lag 22: OR = 1.005; 95% CI = 1.002–1.007; *p* < 0.01), and the PSW (day lag 4: OR = 1.004; 95% CI = 1.001–1.006; *p* < 0.01) region was closer to that of the national trend. The strongest association for the SD of the temperature was observed in the LMW and significant for all 30-day lags (day lag 0: OR = 1.038; 95% CI = 1.026–1.049; *p* < 0.01), followed by the UMW (day lag 7: OR = 1.030, 95% CI = 1.018–1.042, *p* < 0.01) and NE (day lag 6: OR = 1.032; 95% CI = 1.023–1.041; *p* < 0.01). For the RH, the strongest significant association was seen in the SE zone (day lags 13, 24, 25: OR = 1.001; 95% CI = 1.000–1.002, *p* < 0.05). Interestingly, there were more regional variations in the association between the SD of the temperature and MS clinic visit risk compared to the other variables. Finally, for the temperature–RH interactions, the strongest association was seen in the PNW (day lag 0: OR = 1.000; 95% CI = 1.000–1.000, *p* < 0.01). Of note, none of the four meteorological conditions showed significant associations with a clinic visit risk in the ST region on any of the 30-day lags, diverging from the nationally observed trend.

Like with the initial univariate analyses, the regional variations in association between the four meteorological variables and MS clinic visit risk were also seen with multivariate modeling (Table 5). Like with the univariate analyses, all the variables were significant throughout the 30-day lag period nationally. First, the association between the ambient temperature and MS clinic visit risk varied across regions–while many zones showed associations comparable to that of the national level, other zones were more variable. The weakest associations were observed in the SE (day lag 0: OR = 0.973, 95% CI = 0.959–0.987; *p* < 0.01), followed by the DSW (day lag 30: OR = 0.979, 95% CI = 0.963–0.995, *p* < 0.01) and LMW zones (day lag 14: OR = 0.979, 95% CI 0.967–0.992, *p* < 0.01). Interestingly, in the LMW, UMW, NE, and DSW regions, the SD of the temperature showed a stronger association with MS clinic visits than the national trend on several days. For example, associations stronger than the national level were observed in the LMW on the 0-, 7-, 21- and 28-day lags (OR = 1.025, 1.02, 1.026, 1.025, and 1.026, respectively; *p* < 0.01). A similar strength of this association was seen in the DSW (day lag 0: OR = 1.026; 95% CI = 1.009–1.044; *p* < 0.01), but interestingly, this association became insignificant for the rest of the time lags.

A similar trend was observed in the NE, but its peak was weaker than that in the LMW (day lag 21: OR = 1.017, 95% CI = 1.009–1.026, *p* < 0.01). Next, for the association between the RH and clinic visit risk, the strongest association was observed in the ST zone (day lag: OR = 1.004, 95% CI = 0.999–1.009, *p* < 0.05) and the weakest in the SE (day lag 0: OR = 0.994, 95% CI = 0.990–0.997, *p* < 0.01). Finally, the association between the temperature–RH interactions and MS clinic visits was significant for all lags in the LMW only. It was significant for the 0-, 1-, and 7-day lags in SE, PSW, and NE regions. This association was also significant in the DSW region on the 21- and 28-day lags. These associations between the temperature–RH interactions and MS visits were weakly positive at similar magnitudes. The seasonality was also associated with MS visits in the NE (OR = 1.125; 95% CI 1.079–1.174; *p* < 0.01), LMW (OR = 1.10; 95% CI 1.038–1.168); *p* < 0.001), PSW (OR = 1.096; 95% CI 1.024–1.173; *p* < 0.01), and UMW (OR = 1.08; 95% CI 1.019–1.152; *p* < 0.01) regions. In the NE, MS visits were 12.5% more likely to occur in the spring, summer, and fall compared to the winter (November–February).

## 4. Discussion

The findings from our analyses suggest significant associations between temperature, temperature variation (SD of temperature), RH, and temperature-RH interaction with MS visit risk. Across the US, the association between MS clinic visits and ambient temperature was positive in the univariate analysis, but it became inverse when its interaction with RH and other meteorological conditions were introduced in the multivariate analysis. This suggests that while temperature alone is a risk factor of MS clinic visit, its interaction with RH, a proxy of heat index, is a stronger risk factor of MS clinic visit than exposure to ambient temperature along. The magnitude of these associations varied significantly when examined across domestic climate regions, although regardless of stratification, the association between SD of temperature and MS visit risk accounted for the highest magnitude single-day increase in risk at both the national (1.2% increased likelihood) and regional (2.6% increased likelihood, DSW region) levels. While the findings on the association between ambient temperature and MS have are consistent with a majority of studies that report a positive relationships between temperature and MS [7,8,14,22].

Unlikely other meteorological conditions, our research suggests that RH is a protective factor and reduces the risk of MS clinic visit. This finding is consistent with some of the studies and inconsistent with others. For examples, a Brazilian study found a positive correlation between RH and male MS admissions (r = 0.29, *p* < 0.01), while the correlation in females was insignificant (r = 0.04, *p* > 0.05) [23]. Further confusing the literature, an Iranian [17] and an Italian [24] study reported insignificant relationships between RH and MS. Likewise, another retrospective Irish study (1981–1985) of 87 patients with MS found that while frequency of exacerbations was not related to RH (r = 0.11, *p* > 0.05), the average duration of an exacerbation was inversely linked to RH (r = −0.55, *p* < 0.05), similar to our findings [14].

Finally, our study is among the first to report that exposure to temperature variation (SD of temperature) positively associated with MS risk. In our analyses, temperature variation was the strongest predictor of visit risk among all variables, both at the national and regional level. While only a handful of studies have examined the link between temperature variation and MS, similar trends have been reported in these studies. However, comparisons are difficult to make given that ours is the first study to examine this via SD of temperature; in prior studies, diurnal temperature range (DTR), a measure of temperature change as a range (e.g., difference between the maximum and minimum daily values), was instead used as a metric of temperature variation. For example, a Canadian study of 51 patients with known MS from 1950–1953 reported that DTR was positively correlated with for MS exacerbation risk (r = 0.16, *p* = 0.03), suggesting that an increased DTR led to a higher likelihood for symptom occurrence [25]. Furthermore, a Korean study of 1265 MS emergency department visits from 2008-2014 found that increased DTR was significantly associated with increased risk for MS clinic visit (8.81% change in OR per 1 °C increase in the DTR; 95% CI 3.46%–14.44%; *p* < 0.05) [26]. Providing further credence to this relationship, the Irish study above reported that as daily maximum temperature (r = 0.85, *p* < 0.001) or minimum temperature (r = 0.78, *p* < 0.01) reached more extreme levels, duration of MS exacerbations increased, also suggesting that temperature range relates to risk for MS [14]. There is biologic plausibility that temperature variation is associated with MS risk, with the most cited underlying mechanism being dysfunctional thermoregulation (demonstrable loss of thermoregulation, such as sweating mechanics) in patients with MS, as a result of demyelinating brain lesions encompassing temperature regulatory areas of the brain [1,27].

Less commonly noted is activation of mast cells starting within the respiratory tract due to exposure to sudden temperature change leading to worsened neurological symptoms [28], a process similar to that which underlies asthma exacerbations as a result of sudden temperature change [29,30,31,32].

Biologic plausibility also exists for the variability in regional relationships found in our study. First, population-level differences exist—health demographics vary across regions, and may factor into heat sensitivity. For example, certain patient populations have been identified to be more sensitive to heat-related injury (e.g., elderly, females, and patients with decreased mobility or dementia, patients on medications that affect thermoregulation (e.g., diuretics or anticholinergics), and patients with disorders that compromise thermoregulation (e.g., obesity, hypertension, pulmonary disease, diabetes)) [33,34]. Also, individual-level differences may also explain these differences, such as individual climate adaptability to one’s surrounding environment [35]. A Japanese study found that men who lived in hot subtropical zone and moved to colder temperate zones showed superior signs of heat adaptation than those who lived in the temperate region alone (e.g., less skinfold thickness (e.g., upper arm—5.3 ± 2.3 vs. 7.7 ± 3.2mm, *p* < 0.001) and more effective sweating with less salt wasting (0.022 ± 0.004 vs. 0.029 ± 0.008 mEq/l, *p* < 0.05)) [36]. In a similar study, Thai individuals who experience consistently hot and humid climate year-round showed identical differences when compared to Japanese adults who experience variable seasonality as the year goes by [35]. In our study, SD of temperature increased MS visit risk most strongly within the LMW, UMW, NE, and DSW regions, and this may be because subjects from these regions are less tolerant to variable temperature due to a combination of the above explanations (co-morbidities, age, lack of climate adaptability due to less local seasonality). These findings may also explain why some studies on MS that incorporated seasonal analyses reported that the highest frequency of symptom flares occurred during the warmest and coldest months of the year [37], while other studies found that seasonality was not related to symptoms and that severity or frequency of symptoms occurred at a comparable level year-round [38,39]. While climate adaptability can only be improved upon with more time spent in a given area, an Italian study noted that climate susceptibility (‘meteorosensitivity’) can be improved upon with specific behavioral changes. Thus, the latent effects of ambient meteorological conditions on risk for MS may be partially modifiable [40]. In our study, SD of temperature increased MS visit risk most strongly within the LMW, UMW, NE, and DSW regions. This may be because subjects from these regions are less tolerant to variable temperature due to a combination of individual factors (e.g., presence of co-morbidities, age, ethnicity/race/genetic factors, population health disparities [e.g., care access, health literacy, illness perception] as well as environmental factors (lack of climate adaptability due to less local seasonality) [41].

**Figure 4:**
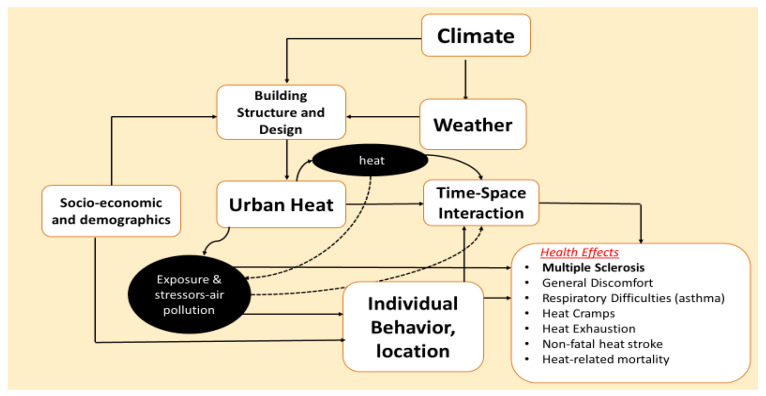
Conceptual framework of the association between the environmental factors and MS.

As with all studies, our findings must be considered in light of its limitations. First, we used ICD-9 codes to assess MS visits, which are susceptible to subjective errors—the accuracy of the diagnosis of MS cannot be ensured given the potential for variable clinical expertise. Second, we were unable to discern if patients were presenting to clinic for an exacerbation or for a routine scheduled visit–as such, we can only focus on associations with environmental factors at the overall disease level. Third, all MS cases were treated in the same way, as we did not have access data on the severity, measured by disability scale, and duration of the disease. Since we did not have access to treatment and prescription data, we could not assess whether MS treatment mediates the role of meteorological conditions in MS. Fourth, A major strength of our study was the availability of a large dataset from which to examine risk for MS. However, the use of VA data greatly limits generalizability given these data represent mostly elderly white males. Issues with generalizability include our study population being predominantly male (while MS tends to affect females disproportionately), as well as the presence of underlying comorbidities (substance abuse, psychiatric disorders, etc.), both of which may have affected our findings. Fortunately, despite demographic differences, our findings were comparable to those of prior studies, with similar associations between select meteorological variables and risk for MS. This suggests that despite demographic differences, similar risk factors are found across varied populations. Besides this, reliance on VA health data required that we used ICD-9 codes to assess MS visits, which are susceptible to subjective errors—the accuracy of the diagnosis of MS cannot be ensured given the potential for variable clinical expertise. Fifth, our environmental data reflected outdoor conditions, while most individuals spend a majority of their time indoors. Finally, using ZIP-codes to stratify patients into regions may be flawed, as some subjects may have been diagnosed at a clinic away from their residence. Despite these limitations, our results identify temperature variation, among other factors, as a strongly associated factor of MS visit risk. The variance in these associations across regions can be explained by the multifaceted nature of the link between environmental sensitivity and MS, some of these pathways being modifiable and thus representing targets for therapeutic improvement in symptomology (Figure 4). These findings may become more relevant given increases in daily temperature variation and heatwave intensity and frequency with global warming. In the meantime, these epidemiological associations can be incorporated into practice, especially for exposure avoidance and/or mitigation in susceptible patients.

## 5. Conclusions

Ambient meteorological conditions are associated with MS symptoms. Our analyses suggest that a sudden increase in ambient temperature and a simultaneous increase in both temperature and relative humidity elevate the risk of MS clinic visits, a proxy measure of adverse MS symptoms. These associations between MS clinic visits and meteorological conditions varied across climate regions, suggesting variations in adaptation to region-specific climatic conditions. Increasing frequency and intensity of extreme weathers patterns, such as heatwaves, drought and hurricanes, are likely to increase the burden of MS disease in the absence of adaptive management strategies. The findings of this research can guide region-specific strategies to manage MS and associated comorbidities, such as providing timely data on heat advisory and sudden changes in local weather patterns to MS patients as well as healthcare providers.

## Figures and Tables

**Table 1:** Region-specific MS clinic visits by US veterans, 2010–2013 (number of MS visits/per 10,000 patient visits).

Region	Mean	CI
DSW	25.4	9.7–41.1
LMW	15.1	11.2–18.9
NE	64.0	−16.7–144.8
PNW	67.6	−24.0–159.2
PSW	25.2	3.1–47.2
SE	41.7	−10.3–93.7
ST	46.8	−19.0–112.5
UMW	24.6	13.4–35.8
Total	39.4	17.6–61.1

**Table 2:** Monthly distribution MS clinic visits by US veterans, 2010–2013 in the US regions (% of total MS visit in the region).

Month	DSW	LMW	NE	PNW	PSW	SE	ST	UMW	Total
1	8.2	7.7	7.9	8.1	8.3	7.8	8.6	8.0	8.0
2	7.9	7.5	7.4	7.7	7.9	8.0	7.8	7.6	7.7
3	8.9	8.8	9.0	8.8	9.1	8.9	9.0	8.9	8.9
4	8.3	8.6	8.6	8.3	8.6	8.2	8.4	8.5	8.5
5	8.4	8.6	8.8	8.7	8.7	8.3	8.4	8.8	8.6
6	8.5	8.5	8.5	8.5	8.5	8.3	8.3	8.4	8.4
7	8.3	8.4	8.3	8.0	8.3	8.3	8.0	8.4	8.3
8	9.2	9.0	8.6	9.0	8.7	9.0	8.8	8.9	8.8
9	8.3	8.4	8.4	8.2	8.1	8.3	8.1	8.2	8.3
10	8.6	8.9	8.8	8.8	8.4	8.8	8.5	8.6	8.7
11	8.0	8.1	8.1	8.2	7.9	8.3	7.9	8.0	8.1
12	7.6	7.4	7.6	7.8	7.7	7.7	8.0	7.8	7.7
Total	100 (53,191)	100 (54,100)	100 (119,781)	100 (44,443)	100 (52,531)	100 (56,871)	100 (55,128)	100 (94,030)	100 (530,075)

**Table 3:** Monthly average temperature (°C) in the United States and US regions by month, 2009–2014 (standard deviation in parenthesis).

Month	US	DSW	LMW	NE	PNW	PSW	SE	ST	UMW
Jan	1.5 (9.0)	4.9 (7.8)	−2.1 (6.9)	−2.1 (6.5)	1.4 (6.6)	9.4 (5.2)	5.1 (6.8)	14.7 (6.3)	−6.3 (7.2)
Feb	3.2 (8.6)	6.5 (8.6)	0.1 (6.8)	−0.3 (5.5)	2.2 (6.1)	9.9 (4.6)	7.2 (6.2)	16.2 (5.8)	−4.6 (6.5)
Mar	7.9 (7.8)	11.6 (7.4)	6.8 (7.0)	4.3 (6.1)	5.3 (4.8)	12.6 (4.6)	11.6 (5.9)	18.2 (4.6)	1.8 (7.2)
Apr	12.9 (6.7)	15.9 (7.3)	12.9 (5.4)	10.4 (5.2)	8.1 (4.3)	15.0 (5.4)	16.9 (4.4)	21.9 (3.3)	7.9 (5.4)
May	17.7 (5.9)	20.0 (6.6)	18.3 (5.0)	16.0 (4.6)	11.9 (4.2)	18.5 (6.0)	21.0 (4.0)	24.6 (2.5)	14.5 (5.3)
Jun	22.0 (5.3)	25.4 (5.1)	23.4 (3.6)	20.1 (4.0)	15.5 (4.0)	21.8 (6.9)	25.2 (2.8)	27.1 (1.7)	19.2 (4.0)
Jul	24.0 (4.5)	26.3 (4.5)	24.9 (3.7)	22.9 (3.5)	19.7 (4.5)	23.9 (7.1)	25.8 (2.7)	27.5 (1.6)	21.9 (3.5)
Aug	23.3 (4.6)	26.4 (5.0)	23.9 (3.3)	21.5 (3.1)	19.2 (4.0)	23.6 (6.6)	25.5 (2.7)	27.8 (1.6)	20.9 (3.1)
Sep	19.8 (5.3)	22.4 (5.5)	19.2 (4.2)	17.8 (4.3)	16.2 (3.8)	22.2 (5.8)	22.4 (3.5)	26.5 (2.1)	16.3 (4.3)
Oct	14.0 (6.2)	16.3 (6.9)	12.7 (4.9)	12.2 (4.7)	10.0 (4.5)	17.4 (4.9)	16.3 (4.7)	23.1 (4.1)	9.7 (4.9)
Nov	8.2 (7.0)	10.2 (7.5)	6.4 (5.7)	6.2 (5.0)	4.6 (6.0)	12.6 (4.8)	10.5 (5.2)	18.7 (5.2)	3.1 (5.9)
Dec	3.6 (8.1)	5.3 (8.2)	0.6 (6.1)	1.5 (5.6)	0.9 (7.0)	8.9 (5.0)	7.1 (5.9)	16.4 (6.1)	−3.0 (6.6)
Total	13.2 (10.4)	16.0 (10.4)	12.3 (10.8)	10.9 (9.8)	9.6 (8.3)	16.4 (7.8)	16.3 (8.8)	21.9 (6.2)	8.5 (11.2)
N	2,249,597	260,647	296,187	529,893	155,558	220,850	273,826	164,316	348,320

**Table 4:** Monthly average relative humidity (%) in the United States and US regions by month, 2009–2014 (standard deviation; number of observations in parenthesis).

Month	US	DSW	LMW	NE	PNW	PSW	SE	ST	UMW
Jan	69.7 (16.0)	61.8 (17.4)	71.0 (13.4)	69.5 (14.0)	79.8 (13.1)	57.9 (22.5)	70.8 (15.2)	73.5 (12.8)	75.2 (10.0)
Feb	69.4 (15.3)	63.6 (18.5)	71.2 (12.8)	67.6 (14.0)	76.6 (12.5)	61.0 (20.8)	70.2 (14.1)	73.9 (12.2)	74.6 (10.1)
Mar	65.6 (16.8)	57.7 (19.9)	67.9 (13.5)	64.9 (15.9)	71.8 (14.7)	55.8 (23.1)	68.6 (14.3)	70.1 (11.2)	69.5 (13.3)
Apr	63.1 (17.3)	56.8 (20.6)	64.5 (14.5)	63.5 (15.9)	66.3 (16.2)	51.0 (23.7)	67.2 (12.9)	71.4 (9.5)	64.8 (14.8)
May	66.2 (17.6)	57.8 (20.6)	69.0 (13.3)	71.9 (13.9)	62.8 (17.2)	47.2 (24.8)	72.9 (10.7)	72.8 (8.7)	66.3 (13.8)
Jun	67.3 (17.2)	55.0 (19.7)	70.5 (10.8)	74.1 (10.8)	62.4 (18.0)	46.9 (25.9)	73.1 (9.4)	75.0 (7.0)	70.4 (11.8)
Jul	68.1 (15.6)	58.5 (16.1)	70.6 (10.8)	73.8 (10.2)	56.1 (19.7)	51.8 (21.9)	75.4 (8.8)	76.9 (6.7)	70.3 (10.5)
Aug	69.2 (15.8)	55.5 (14.2)	72.1 (10.5)	76.9 (8.6)	57.2 (19.7)	51.9 (21.9)	76.1 (8.8)	77.3 (6.4)	72.4 (11.2)
Sep	70.1 (16.1)	60.6 (17.8)	72.1 (11.5)	77.2 (9.6)	60.2 (20.0)	51.7 (21.6)	75.8 (10.0)	77.9 (6.9)	72.3 (12.3)
Oct	69.6 (15.8)	61.0 (16.9)	69.2 (13.1)	75.3 (11.3)	70.5 (17.1)	53.3 (22.3)	73.6 (11.7)	73.8 (9.5)	72.4 (12.4)
Nov	68.7 (15.4)	61.1 (16.8)	68.8 (12.7)	69.9 (13.2)	76.2 (14.8)	56.3 (22.2)	71.8 (12.3)	74.4 (10.1)	71.7 (11.8)
Dec	74.3 (14.1)	68.3 (16.1)	76.6 (11.7)	73.8 (12.7)	80.1 (12.7)	64.6 (20.3)	76.3 (12.3)	76.6 (11.1)	78.1 (9.9)
Total	68.4 (16.3)	59.8 (18.3)	70.3 (12.8)	71.6 (13.4)	68.3 (18.5)	54.1 (23.2)	72.7 (12.2)	74.5 (9.8)	71.5 (12.4)
N	2,249,597	260,647	296,187	529,893	155,558	220,850	273,826	164,316	348,320

**Table 5:** Region-specific associations between MS clinic/hospital visits by US veterans and daily lagged meteorological conditions in the United States, 2010–2013 (odds ratio; 95% confidence intervals in parenthesis).

Variable Name	Time Lag (Day)					
	0	1	7	14	21	28
**United States (US)**						
Ambient temperature (°C)	0.994 ***	0.993 ***	0.995 ***	0.996 ***	0.996 **	0.996 **
	(0.991–0.998)	(0.990–0.997)	(0.991–0.998)	(0.992–0.999)	(0.993–0.999)	(0.993–1.000)
Standard deviation of temperature (°C)	1.012 ***	1.008 ***	1.011 ***	1.005 ***	1.009 ***	1.008 ***
	(1.008–1.017)	(1.004–1.012)	(1.007–1.015)	(1.001–1.009)	(1.005–1.012)	(1.004–1.012)
Relative humidity (%)	0.999 ***	0.998 ***	0.998 ***	0.999 ***	0.999 ***	0.999 ***
	(0.998–1.000)	(0.997–0.999)	(0.997–0.999)	(0.998–0.999)	(0.998–0.999)	(0.998–0.999)
Temperature X relative humidity	1.000 ***	1.000 ***	1.000 ***	1.000 ***	1.000 ***	1.000 **
	(1.000–1.000)	(1.000–1.000)	(1.000–1.000)	(1.000–1.000)	(1.000–1.000)	(1.000–1.000)
Season (0 = Nov to Feb.; 1 otherwise)	1.079 ***	1.083 ***	1.084 ***	1.086 ***	1.087 ***	1.087 ***
	(1.056–1.102)	(1.061–1.107)	(1.061–1.107)	(1.063–1.109)	(1.064–1.110)	(1.064–1.110)
Observations	1,059,723	1,059,391	1,058,304	1,057,287	1,055,302	1,059,006
**Desert Southwest (DSW)**						
Ambient temperature (°C)	1.003	0.999	0.999	0.989	0.981 ***	0.979 **
	(0.993–1.012)	(0.989–1.008)	(0.983–1.014)	(0.972–1.007)	(0.967–0.994)	(0.963–0.995)
Standard deviation of temperature (°C)	1.026 ***	1.012	1.006	0.998	1.001	0.998
	(1.009–1.044)	(0.997–1.026)	(0.993–1.018)	(0.986–1.011)	(0.988–1.013)	(0.985–1.011)
Relative humidity (%)	1	0.998	0.995 **	0.995 **	0.995 ***	0.995 **
	(0.997–1.003)	(0.995–1.001)	(0.991–0.999)	(0.991–0.999)	(0.991–0.998)	(0.991–0.999)
Temperature X relative humidity	1	1	1	1	1.000 ***	1.000 **
	(1.000–1.000)	(1.000–1.000)	(1.000–1.000)	(1.000–1.000)	(1.000–1.000)	(1.000–1.001)
Season (0 = Nov to Feb.; 1 otherwise)	1.01	1.021	1.019	1.051	1.072 *	1.081 **
	(0.940–1.085)	(0.951–1.097)	(0.946–1.097)	(0.975–1.133)	(0.997–1.152)	(1.007–1.161)
Observations	106,376	106,377	106,383	106,267	106,216	106,383
**Lower Midwest (LMW)**						
Ambient temperature (°C)	0.987 **	0.983 ***	0.983 ***	0.979 ***	0.981 ***	0.982 ***
	(0.976–0.997)	(0.972–0.994)	(0.971–0.994)	(0.967–0.992)	(0.970–0.992)	(0.973–0.991)
Standard deviation of temperature (°C)	1.025 ***	1.020 ***	1.026 ***	1.014 **	1.025 ***	1.026 ***
	(1.012–1.038)	(1.007–1.034)	(1.015–1.038)	(1.002–1.026)	(1.013–1.036)	(1.015–1.038)
Relative humidity (%)	0.997 ***	0.996 ***	0.995 ***	0.996 ***	0.996 ***	0.996 ***
	(0.994–0.999)	(0.993–0.998)	(0.993–0.998)	(0.994–0.999)	(0.993–0.998)	(0.994–0.998)
Temperature X relative humidity	1.000 ***	1.000 ***	1.000 ***	1.000 ***	1.000 ***	1.000 ***
	(1.000–1.000)	(1.000–1.000)	(1.000–1.000)	(1.000–1.000)	(1.000–1.000)	(1.000–1.000)
Season (0 = Nov to Feb.; 1 otherwise)	1.101 ***	1.114 ***	1.115 ***	1.131 ***	1.121 ***	1.137 ***
	(1.038–1.168)	(1.050–1.181)	(1.051–1.182)	(1.067–1.199)	(1.060–1.185)	(1.073–1.204)
Observations	108,199	108,199	108,173	108,196	108,191	108,172
**Northeast (NE)**						
Ambient temperature (°C)	0.986 ***	0.989 **	0.996	0.994 **	0.997	0.997
	(0.977–0.994)	(0.980–0.998)	(0.991–1.001)	(0.988–1.000)	(0.991–1.003)	(0.991–1.004)
Standard deviation of temperature (°C)	1.014 ***	1.008 *	1.014 ***	1.009 **	1.017 ***	1.014 ***
	(1.005–1.024)	(0.999–1.017)	(1.006–1.023)	(1.000–1.018)	(1.009–1.026)	(1.005–1.023)
Relative humidity (%)	0.998 ***	0.998 **	0.999 *	0.998 **	0.999 *	0.998 ***
	(0.996–0.999)	(0.997–1.000)	(0.997–1.000)	(0.997–1.000)	(0.997–1.000)	(0.996–0.999)
Temperature X relative humidity	1.000 ***	1.000 **	1	1.000 **	1	1
	(1.000–1.000)	(1.000–1.000)	(1.000–1.000)	(1.000–1.000)	(1.000–1.000)	(1.000–1.000)
Season (0 = Nov to Feb.; 1 otherwise)	1.125 ***	1.131 ***	1.122 ***	1.119 ***	1.114 ***	1.107 ***
	(1.079–1.174)	(1.085–1.180)	(1.075–1.171)	(1.072–1.169)	(1.068–1.163)	(1.061–1.155)
Observations	239,452	239,111	239,376	239,482	239,495	239,494
**Pacific Northwest (PNW)**						
Ambient temperature (°C)	1	0.999	1.004	0.999	1	0.991
	(0.986–1.014)	(0.987–1.011)	(0.992–1.016)	(0.983–1.014)	(0.984–1.016)	(0.976–1.007)
Standard deviation of temperature (°C)	0.999	1.004	0.992	0.99	0.989*	0.997
	(0.981–1.018)	(0.988–1.020)	(0.980–1.005)	(0.977–1.003)	(0.976–1.002)	(0.984–1.010)
Relative humidity (%)	1	1	1.001	0.998	1	0.999
	(0.997–1.003)	(0.998–1.003)	(0.999–1.004)	(0.995–1.001)	(0.997–1.003)	(0.996–1.002)
Temperature X relative humidity	1	1	1	1	1	1
	(1.000–1.000)	(1.000–1.000)	(1.000–1.000)	(1.000–1.000)	(1.000–1.000)	(1.000–1.000)
Season (0 = Nov to Feb.; 1 otherwise)	1.048	1.043	1.03	1.051	1.066	1.055
	(0.960–1.143)	(0.956–1.137)	(0.944–1.123)	(0.967–1.142)	(0.975–1.165)	(0.966–1.152)
Observations	88,886	88,886	88,874	88,886	88,885	88,451
**Pacific Southwest (PSW)**						
Ambient temperature (°C)	0.995	0.992 **	0.994 *	0.999	0.990 **	0.999
	(0.986–1.004)	(0.984–1.000)	(0.988–1.000)	(0.991–1.007)	(0.981–1.000)	(0.987–1.011)
Standard deviation of temperature (°C)	1.015 *	1.015 *	1.013 *	1.002	1.006	0.999
	(0.999–1.031)	(0.999–1.031)	(0.999–1.027)	(0.988–1.016)	(0.992–1.020)	(0.985–1.013)
Relative humidity (%)	0.999	0.997 *	0.998	0.999	0.996 **	0.998
	(0.996–1.002)	(0.995–1.000)	(0.996–1.001)	(0.997–1.002)	(0.993–0.999)	(0.995–1.002)
Temperature X relative humidity	1	1.000 ***	1.000 *	1	1	1
	(1.000–1.000)	(1.000–1.000)	(1.000–1.000)	(1.000–1.000)	(1.000–1.000)	(1.000–1.000)
Season (0 = Nov to Feb.; 1 otherwise)	1.096 ***	1.088 **	1.102 ***	1.096 ***	1.122 ***	1.113 ***
	(1.024–1.173)	(1.018–1.163)	(1.031–1.179)	(1.023–1.175)	(1.047–1.203)	(1.038–1.194)
Observations	105,012	105,029	103,864	105,061	105,061	104,490
**Southeast (SE)**						
Ambient temperature (°C)	0.973 ***	0.982 ***	0.994	1.001	0.998	1
	(0.959–0.987)	(0.969–0.995)	(0.984–1.004)	(0.993–1.008)	(0.990–1.005)	(0.992–1.007)
Standard deviation of temperature (°C)	1.012 *	1.002	1.004	1.007	1.005	1.009
	(0.998–1.026)	(0.989–1.015)	(0.992–1.017)	(0.994–1.019)	(0.993–1.017)	(0.997–1.021)
Relative humidity (%)	0.994 ***	0.995 ***	0.997 **	1	0.999	1
	(0.990–0.997)	(0.991–0.998)	(0.994–1.000)	(0.997–1.002)	(0.997–1.001)	(0.997–1.002)
Temperature X relative humidity	1.000 ***	1.000 ***	1.000 **	1	1	1
	(1.000–1.001)	(1.000–1.000)	(1.000–1.000)	(1.000–1.000)	(1.000–1.000)	(1.000–1.000)
Season (0 = Nov to Feb.; 1 otherwise)	1.034	1.036	1.015	1.017	1.025	1.031
	(0.970–1.103)	(0.972–1.104)	(0.953–1.081)	(0.953–1.086)	(0.963–1.090)	(0.969–1.096)
Observations	113,682	113,709	113,705	111,316	113,704	113,736
**Subtropical (ST)**						
Ambient temperature (°C)	1.017	1.008	1.001	0.99	1.012	1.002
	(0.988–1.047)	(0.983–1.034)	(0.980–1.022)	(0.970–1.010)	(0.996–1.028)	(0.986–1.019)
Standard deviation of temperature (°C)	1	0.996	1.008	1.008	1.008	1.015
	(0.981–1.019)	(0.979–1.013)	(0.992–1.025)	(0.991–1.026)	(0.989–1.027)	(0.996–1.034)
Relative humidity (%)	1.005	1.003	1.002	0.999	1.004 *	1.002
	(0.996–1.013)	(0.996–1.010)	(0.996–1.007)	(0.994–1.004)	(0.999–1.009)	(0.998–1.005)
Temperature X relative humidity	1	1	1	1	1	1
	(0.999–1.000)	(0.999–1.000)	(1.000–1.000)	(1.000–1.000)	(1.000–1.000)	(1.000–1.000)
Season (0 = Nov to Feb.; 1 otherwise)	1.044	1.044	1.049	1.036	1.037	1.041
	(0.958–1.137)	(0.961–1.135)	(0.970–1.134)	(0.959–1.120)	(0.964–1.116)	(0.965–1.122)
Observations	110,254	110,233	110,075	110,244	110,248	110,232
**Upper Midwest (UMW)**						
Ambient temperature (°C)	0.988 **	0.985 **	0.985 **	0.993	1	1.006
	(0.976–1.000)	(0.973–0.997)	(0.972–0.998)	(0.981–1.005)	(0.989–1.012)	(0.995–1.017)
Standard deviation of temperature (°C)	1.017 ***	1.013 **	1.023 ***	1.011 *	1.018 ***	1.012 **
	(1.004–1.029)	(1.000–1.025)	(1.011–1.035)	(1.000–1.023)	(1.006–1.030)	(1.001–1.024)
Relative humidity (%)	0.997 **	0.997 **	0.997 **	0.998	0.999	0.999
	(0.995–1.000)	(0.994–0.999)	(0.995–1.000)	(0.996–1.001)	(0.997–1.001)	(0.997–1.001)
Temperature X relative humidity	1.000 **	1.000 **	1.000 **	1	1	1
	(1.000–1.000)	(1.000–1.000)	(1.000–1.000)	(1.000–1.000)	(1.000–1.000)	(1.000–1.000)
Season (0 = Nov to Feb.; 1 otherwise)	1.083 **	1.098 ***	1.095 ***	1.094 ***	1.076 **	1.089 ***
	(1.019–1.152)	(1.033–1.167)	(1.030–1.163)	(1.031–1.162)	(1.012–1.144)	(1.025–1.156)
Observations	187,862	187,847	187,854	187,835	183,502	188,048

Robust 95% confidence interval in parentheses; *** *p* < 0.01, ** *p* < 0.05, and * *p* < 0.1.

## Data Availability

Data used in the research were confidential and cannot be shared outside our research team as required by the Miami VA Institutional Review Board.

## Supplementary Materials

The following are available online at https://www.mdpi.com/article/10.3390/ijerph18115962/s1, Table S1: Region specific associations between MS clinic/hospital visits by the US veterans and daily lagged meteorological conditions in the United States, 2010–2013—a univariate analysis adjusted for weekend/weekday effect.
