# Supplementary material for: Heat Exposure and Multiple Sclerosis—A Regional and Temporal Analysis
Source: Int J Environ Res Public Health. 2021 Jun 2;18(11):5962. doi: 10.3390/ijerph18115962 (PMC8199586; doi:10.3390/ijerph18115962)
Supplement: Supplementary file 1 [file ijerph-18-05962-s001.zip › ijerph-1216874-supplementary.pdf]

Heat Exposure and Multiple Sclerosis – A Regional and Temporal Analysis

Supplement Online Material

Table S1: Region specific associations between MS clinic/hospital visits by the US veterans and daily lagged meteorological conditions in the United States, 2010-2013 – a univariate analysis adjusted for weekend/weekday effect. Odds ratio (95% confidence interval in parenthesis); * p ≤ 0.05, ** p ≤ 0.01

Lag (day)	Temperature (° C)	Standard deviation of hourly temperature (° C)	Relative Humidity (%)	Temperature X Relative humidity
**United States**				
0	1.003** (1.002 - 1.004)	1.016** (1.012 - 1.019)	0.999** (0.999 - 1.000)	1.000** (1.000 - 1.000)
1	1.003** (1.002 - 1.004)	1.012** (1.009 - 1.016)	0.999** (0.999 - 1.000)	1.000** (1.000 - 1.000)
2	1.003** (1.002 - 1.004)	1.009** (1.005 - 1.013)	0.999** (0.999 - 0.999)	1.000** (1.000 - 1.000)
3	1.003** (1.002 - 1.004)	1.009** (1.005 - 1.013)	0.999** (0.999 - 0.999)	1.000** (1.000 - 1.000)
4	1.003** (1.002 - 1.004)	1.011** (1.007 - 1.015)	0.999** (0.999 - 0.999)	1.000** (1.000 - 1.000)
5	1.003** (1.002 - 1.004)	1.013** (1.009 - 1.017)	0.999** (0.999 - 0.999)	1.000** (1.000 - 1.000)
6	1.003** (1.002 - 1.004)	1.016** (1.012 - 1.019)	0.999** (0.999 - 0.999)	1.000** (1.000 - 1.000)
7	1.003** (1.002 - 1.004)	1.016** (1.012 - 1.019)	0.999** (0.999 - 1.000)	1.000** (1.000 - 1.000)
8	1.003** (1.002 - 1.004)	1.012** (1.009 - 1.016)	0.999** (0.999 - 1.000)	1.000** (1.000 - 1.000)
9	1.003** (1.002 - 1.004)	1.011** (1.007 - 1.015)	0.999** (0.999 - 1.000)	1.000** (1.000 - 1.000)
10	1.003** (1.002 - 1.004)	1.010** (1.006 - 1.014)	0.999** (0.999 - 1.000)	1.000** (1.000 - 1.000)
11	1.003** (1.002 - 1.004)	1.009** (1.005 - 1.013)	0.999** (0.999 - 1.000)	1.000** (1.000 - 1.000)
12	1.003** (1.002 - 1.004)	1.010** (1.006 - 1.013)	0.999** (0.999 - 1.000)	1.000** (1.000 - 1.000)
13	1.003** (1.002 - 1.004)	1.011** (1.007 - 1.015)	1.000** (0.999 - 1.000)	1.000** (1.000 - 1.000)
14	1.003** (1.002 - 1.004)	1.009** (1.006 - 1.013)	0.999** (0.999 - 1.000)	1.000** (1.000 - 1.000)
15	1.003** (1.002 - 1.004)	1.011** (1.007 - 1.015)	0.999** (0.999 - 1.000)	1.000** (1.000 - 1.000)
16	1.003** (1.002 - 1.004)	1.010** (1.007 - 1.014)	0.999** (0.999 - 1.000)	1.000** (1.000 - 1.000)
17	1.003** (1.002 - 1.004)	1.007** (1.003 - 1.011)	0.999** (0.999 - 1.000)	1.000** (1.000 - 1.000)
18	1.003** (1.002 - 1.004)	1.007** (1.003 - 1.011)	0.999** (0.999 - 1.000)	1.000** (1.000 - 1.000)
19	1.003** (1.002 - 1.004)	1.011** (1.008 - 1.015)	0.999** (0.999 - 1.000)	1.000** (1.000 - 1.000)
20	1.003** (1.002 - 1.004)	1.012** (1.009 - 1.016)	0.999** (0.999 - 1.000)	1.000** (1.000 - 1.000)
21	1.003** (1.002 - 1.004)	1.012** (1.009 - 1.016)	0.999** (0.999 - 1.000)	1.000** (1.000 - 1.000)
22	1.003** (1.002 - 1.004)	1.009** (1.005 - 1.013)	0.999** (0.999 - 1.000)	1.000** (1.000 - 1.000)
23	1.003** (1.002 - 1.004)	1.009** (1.005 - 1.013)	0.999** (0.999 - 1.000)	1.000** (1.000 - 1.000)
24	1.003** (1.002 - 1.004)	1.008** (1.005 - 1.012)	1.000* (0.999 - 1.000)	1.000** (1.000 - 1.000)
25	1.003** (1.002 - 1.004)	1.008** (1.004 - 1.012)	0.999** (0.999 - 1.000)	1.000** (1.000 - 1.000)
26	1.003** (1.002 - 1.004)	1.010** (1.006 - 1.014)	0.999** (0.999 - 1.000)	1.000** (1.000 - 1.000)
27	1.003** (1.002 - 1.004)	1.011** (1.007 - 1.015)	0.999** (0.999 - 1.000)	1.000** (1.000 - 1.000)
28	1.003** (1.002 - 1.004)	1.011** (1.008 - 1.015)	0.999** (0.999 - 0.999)	1.000** (1.000 - 1.000)
29	1.003** (1.002 - 1.004)	1.007** (1.004 - 1.011)	0.999** (0.999 - 0.999)	1.000** (1.000 - 1.000)
30	1.003** (1.002 - 1.004)	1.006** (1.002 - 1.010)	0.999** (0.999 - 0.999)	1.000** (1.000 - 1.000)
**Desert Southwest**				
0	1.004** (1.001 - 1.007)	1.018** (1.006 - 1.030)	0.999 (0.998 - 1.000)	1.000** (1.000 - 1.000)
1	1.004** (1.001 - 1.007)	1.014* (1.002 - 1.026)	0.999* (0.997 - 1.000)	1.000** (1.000 - 1.000)

2	1.004** (1.001 - 1.007)	1.009 (0.997 - 1.021)	0.998** (0.997 - 0.999)	1 (1.000 - 1.000)
3	1.004** (1.001 - 1.007)	1.006 (0.995 - 1.018)	0.996** (0.995 - 0.998)	1 (1.000 - 1.000)
4	1.004** (1.001 - 1.008)	1.009 (0.998 - 1.021)	0.996** (0.994 - 0.998)	1 (1.000 - 1.000)
5	1.004** (1.001 - 1.008)	1.005 (0.993 - 1.017)	0.996** (0.995 - 0.998)	1 (1.000 - 1.000)
6	1.004* (1.001 - 1.007)	1.004 (0.992 - 1.015)	0.996** (0.995 - 0.998)	1 (1.000 - 1.000)
7	1.004* (1.000 - 1.007)	1.009 (0.998 - 1.021)	0.996** (0.994 - 0.998)	1 (1.000 - 1.000)
8	1.004* (1.000 - 1.007)	1.007 (0.995 - 1.019)	0.996** (0.994 - 0.998)	1 (1.000 - 1.000)
9	1.004* (1.000 - 1.007)	1.004 (0.992 - 1.015)	0.996** (0.994 - 0.997)	1 (1.000 - 1.000)
10	1.003 (1.000 - 1.007)	1 (0.988 - 1.011)	0.996** (0.994 - 0.998)	1 (1.000 - 1.000)
11	1.003 (1.000 - 1.007)	0.998 (0.987 - 1.010)	0.997** (0.995 - 0.999)	1 (1.000 - 1.000)
12	1.003 (1.000 - 1.007)	0.999 (0.987 - 1.011)	0.998 (0.996 - 1.000)	1 (1.000 - 1.000)
13	1.003 (1.000 - 1.007)	1.003 (0.992 - 1.015)	0.998 (0.996 - 1.000)	1 (1.000 - 1.000)
14	1.003 (1.000 - 1.007)	1 (0.988 - 1.012)	0.998* (0.996 - 1.000)	1 (1.000 - 1.000)
15	1.003 (1.000 - 1.007)	1.007 (0.995 - 1.019)	0.998 (0.996 - 1.000)	1 (1.000 - 1.000)
16	1.003 (1.000 - 1.007)	1.008 (0.997 - 1.020)	0.999 (0.997 - 1.000)	1 (1.000 - 1.000)
17	1.003 (1.000 - 1.007)	1.008 (0.996 - 1.019)	0.999 (0.998 - 1.001)	1 (1.000 - 1.000)
18	1.003 (0.999 - 1.007)	0.999 (0.988 - 1.011)	0.999 (0.998 - 1.001)	1 (1.000 - 1.000)
19	1.003 (0.999 - 1.007)	1.005 (0.994 - 1.017)	1 (0.998 - 1.001)	1 (1.000 - 1.000)
20	1.003 (0.999 - 1.007)	1.006 (0.995 - 1.018)	0.999 (0.998 - 1.001)	1 (1.000 - 1.000)
21	1.003 (0.999 - 1.006)	1.002 (0.990 - 1.013)	0.999 (0.998 - 1.001)	1 (1.000 - 1.000)
22	1.003 (0.999 - 1.006)	1 (0.988 - 1.012)	0.999 (0.998 - 1.001)	1 (1.000 - 1.000)
23	1.003 (0.999 - 1.006)	0.995 (0.983 - 1.006)	1 (0.998 - 1.001)	1 (1.000 - 1.000)
24	1.003 (0.999 - 1.006)	0.997 (0.986 - 1.009)	1 (0.998 - 1.001)	1 (1.000 - 1.000)
25	1.003 (0.999 - 1.006)	0.997 (0.985 - 1.008)	0.999 (0.998 - 1.001)	1 (1.000 - 1.000)
26	1.003 (0.999 - 1.006)	1 (0.988 - 1.011)	1 (0.998 - 1.002)	1 (1.000 - 1.000)
27	1.003 (0.999 - 1.007)	0.995 (0.983 - 1.007)	0.999 (0.997 - 1.001)	1 (1.000 - 1.000)
28	1.003 (0.999 - 1.006)	0.998 (0.987 - 1.010)	1 (0.998 - 1.001)	1 (1.000 - 1.000)
29	1.003 (0.999 - 1.006)	0.997 (0.985 - 1.009)	1 (0.998 - 1.001)	1 (1.000 - 1.000)
30	1.003 (0.999 - 1.006)	0.999 (0.988 - 1.011)	0.999 (0.998 - 1.001)	1 (1.000 - 1.000)
**Lower Midwest**				
0	1.006** (1.004 - 1.008)	1.038** (1.026 - 1.049)	0.997** (0.996 - 0.998)	1.000** (1.000 - 1.000)
1	1.006** (1.004 - 1.008)	1.034** (1.022 - 1.046)	0.997** (0.995 - 0.998)	1.000** (1.000 - 1.000)
2	1.006** (1.003 - 1.008)	1.026** (1.015 - 1.038)	0.997** (0.995 - 0.998)	1.000** (1.000 - 1.000)
3	1.006** (1.003 - 1.008)	1.021** (1.010 - 1.033)	0.996** (0.995 - 0.998)	1.000** (1.000 - 1.000)
4	1.006** (1.003 - 1.008)	1.025** (1.013 - 1.037)	0.996** (0.995 - 0.998)	1.000** (1.000 - 1.000)
5	1.006** (1.003 - 1.008)	1.025** (1.014 - 1.037)	0.997** (0.995 - 0.998)	1.000** (1.000 - 1.000)
6	1.006** (1.003 - 1.008)	1.028** (1.016 - 1.040)	0.997** (0.996 - 0.999)	1.000** (1.000 - 1.000)
7	1.006** (1.003 - 1.008)	1.036** (1.024 - 1.048)	0.998** (0.996 - 0.999)	1.000** (1.000 - 1.000)
8	1.006** (1.003 - 1.008)	1.028** (1.016 - 1.039)	0.998** (0.996 - 0.999)	1.000** (1.000 - 1.000)
9	1.006** (1.003 - 1.008)	1.028** (1.016 - 1.039)	0.998* (0.997 - 1.000)	1.000** (1.000 - 1.000)
10	1.006** (1.003 - 1.008)	1.029** (1.018 - 1.041)	0.998* (0.997 - 1.000)	1.000** (1.000 - 1.000)
11	1.006** (1.003 - 1.008)	1.026** (1.014 - 1.038)	0.999 (0.997 - 1.000)	1.000** (1.000 - 1.000)
12	1.006** (1.003 - 1.008)	1.030** (1.018 - 1.042)	0.998 (0.997 - 1.000)	1.000** (1.000 - 1.000)
13	1.006** (1.003 - 1.008)	1.019** (1.008 - 1.031)	0.999 (0.997 - 1.000)	1.000** (1.000 - 1.000)
14	1.006** (1.003 - 1.008)	1.023** (1.011 - 1.034)	0.999 (0.998 - 1.001)	1.000** (1.000 - 1.000)
15	1.006** (1.003 - 1.008)	1.024** (1.013 - 1.036)	0.999 (0.998 - 1.001)	1.000** (1.000 - 1.000)

16	1.006** (1.003 - 1.008)	1.028** (1.016 - 1.039)	0.999 (0.998 - 1.001)	1.000** (1.000 - 1.000)
17	1.006** (1.003 - 1.008)	1.023** (1.012 - 1.035)	1 (0.998 - 1.001)	1.000** (1.000 - 1.000)
18	1.006** (1.003 - 1.008)	1.023** (1.012 - 1.035)	0.999 (0.998 - 1.001)	1.000** (1.000 - 1.000)
19	1.006** (1.003 - 1.008)	1.021** (1.010 - 1.033)	0.999 (0.997 - 1.000)	1.000** (1.000 - 1.000)
20	1.006** (1.003 - 1.008)	1.027** (1.016 - 1.039)	0.999 (0.997 - 1.000)	1.000** (1.000 - 1.000)
21	1.006** (1.003 - 1.008)	1.032** (1.020 - 1.044)	0.999 (0.997 - 1.000)	1.000** (1.000 - 1.000)
22	1.006** (1.003 - 1.008)	1.025** (1.014 - 1.037)	0.999 (0.997 - 1.000)	1.000** (1.000 - 1.000)
23	1.006** (1.003 - 1.008)	1.025** (1.013 - 1.037)	0.999 (0.998 - 1.001)	1.000** (1.000 - 1.000)
24	1.006** (1.003 - 1.008)	1.027** (1.016 - 1.039)	0.999 (0.998 - 1.001)	1.000** (1.000 - 1.000)
25	1.006** (1.003 - 1.008)	1.022** (1.011 - 1.034)	0.999 (0.998 - 1.001)	1.000** (1.000 - 1.000)
26	1.006** (1.003 - 1.008)	1.021** (1.009 - 1.033)	0.999 (0.997 - 1.000)	1.000** (1.000 - 1.000)
27	1.006** (1.003 - 1.008)	1.018** (1.007 - 1.030)	0.999 (0.998 - 1.000)	1.000** (1.000 - 1.000)
28	1.006** (1.003 - 1.008)	1.031** (1.019 - 1.043)	0.999 (0.998 - 1.000)	1.000** (1.000 - 1.000)
29	1.006** (1.003 - 1.008)	1.028** (1.017 - 1.040)	0.999 (0.998 - 1.000)	1.000** (1.000 - 1.000)
30	1.006** (1.003 - 1.008)	1.026** (1.015 - 1.038)	0.999 (0.998 - 1.000)	1.000** (1.000 - 1.000)
**Northeast**				
0	1.004** (1.002 - 1.006)	1.019** (1.010 - 1.027)	1 (0.999 - 1.001)	1.000** (1.000 - 1.000)
1	1.004** (1.002 - 1.006)	1.013** (1.004 - 1.021)	1 (0.999 - 1.001)	1.000** (1.000 - 1.000)
2	1.004** (1.002 - 1.006)	1.012** (1.004 - 1.021)	1 (0.999 - 1.001)	1.000** (1.000 - 1.000)
3	1.004** (1.002 - 1.006)	1.016** (1.008 - 1.025)	0.999 (0.999 - 1.000)	1.000** (1.000 - 1.000)
4	1.004** (1.002 - 1.006)	1.020** (1.011 - 1.029)	0.999 (0.998 - 1.000)	1.000** (1.000 - 1.000)
5	1.004** (1.002 - 1.006)	1.020** (1.011 - 1.028)	0.999* (0.999 - 1.000)	1.000** (1.000 - 1.000)
6	1.004** (1.002 - 1.006)	1.032** (1.023 - 1.041)	0.999** (0.998 - 1.000)	1.000** (1.000 - 1.000)
7	1.004** (1.002 - 1.006)	1.021** (1.012 - 1.030)	0.999* (0.999 - 1.000)	1.000** (1.000 - 1.000)
8	1.004** (1.002 - 1.006)	1.016** (1.008 - 1.025)	0.999** (0.998 - 1.000)	1.000** (1.000 - 1.000)
9	1.004** (1.002 - 1.006)	1.016** (1.007 - 1.024)	0.999** (0.998 - 1.000)	1.000** (1.000 - 1.000)
10	1.004** (1.002 - 1.006)	1.011** (1.003 - 1.020)	0.999** (0.999 - 1.000)	1.000** (1.000 - 1.000)
11	1.004** (1.002 - 1.006)	1.013** (1.005 - 1.022)	0.999** (0.999 - 1.000)	1.000** (1.000 - 1.000)
12	1.004** (1.002 - 1.006)	1.018** (1.010 - 1.027)	0.999* (0.999 - 1.000)	1.000** (1.000 - 1.000)
13	1.004** (1.002 - 1.006)	1.018** (1.009 - 1.026)	0.999* (0.999 - 1.000)	1.000** (1.000 - 1.000)
14	1.004** (1.002 - 1.006)	1.016** (1.007 - 1.025)	0.999* (0.999 - 1.000)	1.000** (1.000 - 1.000)
15	1.004** (1.002 - 1.006)	1.019** (1.011 - 1.028)	0.999** (0.998 - 1.000)	1.000** (1.000 - 1.000)
16	1.004** (1.002 - 1.006)	1.018** (1.010 - 1.027)	0.999** (0.998 - 1.000)	1.000** (1.000 - 1.000)
17	1.004** (1.002 - 1.006)	1.004 (0.996 - 1.013)	0.999** (0.998 - 0.999)	1.000** (1.000 - 1.000)
18	1.004** (1.002 - 1.006)	1.011** (1.002 - 1.020)	0.999** (0.998 - 0.999)	1.000** (1.000 - 1.000)
19	1.004** (1.002 - 1.006)	1.023** (1.014 - 1.032)	0.999** (0.998 - 0.999)	1.000** (1.000 - 1.000)
20	1.004** (1.002 - 1.006)	1.023** (1.015 - 1.032)	0.999** (0.998 - 0.999)	1.000** (1.000 - 1.000)
21	1.004** (1.002 - 1.006)	1.024** (1.015 - 1.032)	0.999** (0.998 - 0.999)	1.000** (1.000 - 1.000)
22	1.004** (1.002 - 1.006)	1.013** (1.005 - 1.022)	0.999** (0.998 - 0.999)	1.000** (1.000 - 1.000)
23	1.004** (1.002 - 1.006)	1.015** (1.006 - 1.023)	0.998** (0.998 - 0.999)	1.000** (1.000 - 1.000)
24	1.004** (1.003 - 1.006)	1.013** (1.004 - 1.022)	0.999** (0.998 - 0.999)	1.000** (1.000 - 1.000)
25	1.004** (1.002 - 1.006)	1.016** (1.007 - 1.025)	0.998** (0.998 - 0.999)	1.000** (1.000 - 1.000)
26	1.004** (1.002 - 1.006)	1.021** (1.013 - 1.030)	0.998** (0.998 - 0.999)	1.000** (1.000 - 1.000)
27	1.004** (1.002 - 1.006)	1.022** (1.014 - 1.031)	0.998** (0.997 - 0.999)	1.000** (1.000 - 1.000)
28	1.004** (1.002 - 1.006)	1.019** (1.011 - 1.028)	0.998** (0.997 - 0.999)	1.000** (1.000 - 1.000)
29	1.004** (1.002 - 1.006)	1.011** (1.002 - 1.019)	0.998** (0.997 - 0.999)	1.000** (1.000 - 1.000)

30	1.004** (1.002 - 1.006)	1.008 (0.999 - 1.016)	0.998** (0.997 - 0.999)	1.000** (1.000 - 1.000)
**Pacific Northwest**				
0	1.005* (1.000 - 1.009)	1.002 (0.989 - 1.015)	1 (0.999 - 1.001)	1.000** (1.000 - 1.000)
1	1.004* (1.000 - 1.008)	1.006 (0.994 - 1.019)	1 (0.998 - 1.001)	1.000** (1.000 - 1.000)
2	1.004* (1.000 - 1.008)	1.008 (0.996 - 1.021)	0.999 (0.998 - 1.001)	1.000** (1.000 - 1.000)
3	1.004* (1.000 - 1.007)	1.006 (0.993 - 1.018)	1 (0.998 - 1.001)	1.000** (1.000 - 1.000)
4	1.004** (1.001 - 1.008)	1.005 (0.993 - 1.017)	1 (0.998 - 1.001)	1.000** (1.000 - 1.000)
5	1.005** (1.001 - 1.008)	1.007 (0.994 - 1.020)	0.999 (0.997 - 1.001)	1.000** (1.000 - 1.000)
6	1.004** (1.001 - 1.007)	1.005 (0.992 - 1.017)	0.999 (0.998 - 1.001)	1.000** (1.000 - 1.000)
7	1.004** (1.001 - 1.007)	1.004 (0.992 - 1.017)	1 (0.998 - 1.001)	1.000** (1.000 - 1.000)
8	1.004* (1.001 - 1.007)	1.007 (0.995 - 1.020)	1 (0.998 - 1.001)	1.000** (1.000 - 1.000)
9	1.004** (1.001 - 1.007)	1.006 (0.994 - 1.019)	0.999 (0.997 - 1.001)	1.000** (1.000 - 1.000)
10	1.004* (1.001 - 1.007)	1.008 (0.996 - 1.021)	0.999 (0.997 - 1.000)	1.000* (1.000 - 1.000)
11	1.003* (1.000 - 1.006)	1.005 (0.993 - 1.018)	0.998 (0.996 - 1.000)	1 (1.000 - 1.000)
12	1.003 (1.000 - 1.006)	1.005 (0.992 - 1.018)	0.998 (0.996 - 1.000)	1 (1.000 - 1.000)
13	1.003 (1.000 - 1.006)	1.005 (0.992 - 1.018)	0.998* (0.996 - 1.000)	1 (1.000 - 1.000)
14	1.003 (0.999 - 1.006)	0.999 (0.986 - 1.011)	0.997** (0.995 - 1.000)	1 (1.000 - 1.000)
15	1.003 (0.999 - 1.006)	1.002 (0.989 - 1.015)	0.998 (0.996 - 1.000)	1 (1.000 - 1.000)
16	1.003 (0.999 - 1.006)	0.998 (0.986 - 1.011)	0.998 (0.996 - 1.000)	1 (1.000 - 1.000)
17	1.003 (0.999 - 1.006)	1.001 (0.989 - 1.014)	0.999 (0.997 - 1.001)	1 (1.000 - 1.000)
18	1.003 (0.999 - 1.006)	1.003 (0.990 - 1.015)	0.999 (0.997 - 1.001)	1 (1.000 - 1.000)
19	1.003 (0.999 - 1.006)	1 (0.988 - 1.013)	0.999 (0.997 - 1.001)	1 (1.000 - 1.000)
20	1.003 (0.999 - 1.006)	0.999 (0.986 - 1.011)	0.999 (0.998 - 1.001)	1 (1.000 - 1.000)
21	1.003 (1.000 - 1.007)	0.997 (0.984 - 1.009)	1 (0.999 - 1.002)	1 (1.000 - 1.000)
22	1.003 (1.000 - 1.007)	1.001 (0.988 - 1.014)	1.001 (0.999 - 1.002)	1 (1.000 - 1.000)
23	1.003 (0.999 - 1.006)	1.002 (0.990 - 1.015)	1.001 (0.999 - 1.002)	1 (1.000 - 1.000)
24	1.003 (0.999 - 1.006)	1.005 (0.993 - 1.018)	1.001 (1.000 - 1.003)	1.000* (1.000 - 1.000)
25	1.003 (1.000 - 1.007)	1.004 (0.991 - 1.016)	1.001 (0.999 - 1.003)	1.000* (1.000 - 1.000)
26	1.003 (1.000 - 1.007)	1.005 (0.992 - 1.017)	1 (0.999 - 1.002)	1.000* (1.000 - 1.000)
27	1.003 (1.000 - 1.007)	1.002 (0.990 - 1.015)	1 (0.998 - 1.002)	1.000* (1.000 - 1.000)
28	1.003* (1.000 - 1.007)	1.004 (0.991 - 1.017)	1 (0.998 - 1.002)	1.000* (1.000 - 1.000)
29	1.003 (1.000 - 1.006)	1.005 (0.993 - 1.018)	1 (0.998 - 1.002)	1.000* (1.000 - 1.000)
30	1.003 (1.000 - 1.007)	1.003 (0.990 - 1.015)	1 (0.997 - 1.002)	1.000* (1.000 - 1.000)
**Pacific Southwest**				
0	1.003 (0.999 - 1.006)	1.014* (1.001 - 1.027)	1 (0.999 - 1.001)	1.000* (1.000 - 1.000)
1	1.003 (1.000 - 1.006)	1.015* (1.002 - 1.029)	1 (0.999 - 1.000)	1.000** (1.000 - 1.000)
2	1.003 (1.000 - 1.006)	1.018** (1.004 - 1.031)	0.999 (0.999 - 1.000)	1.000** (1.000 - 1.000)
3	1.003 (1.000 - 1.006)	1.019** (1.005 - 1.032)	1 (0.999 - 1.000)	1.000** (1.000 - 1.000)
4	1.004** (1.001 - 1.006)	1.014* (1.000 - 1.027)	0.999 (0.998 - 1.000)	1.000** (1.000 - 1.000)
5	1.003* (1.001 - 1.006)	1.016** (1.003 - 1.030)	0.999 (0.998 - 1.000)	1.000** (1.000 - 1.000)
6	1.003* (1.000 - 1.005)	1.016** (1.003 - 1.030)	1 (0.998 - 1.001)	1.000* (1.000 - 1.000)
7	1.002 (1.000 - 1.005)	1.015* (1.002 - 1.028)	1 (0.999 - 1.001)	1.000** (1.000 - 1.000)
8	1.002 (1.000 - 1.005)	1.01 (0.996 - 1.023)	1 (0.999 - 1.001)	1 (1.000 - 1.000)
9	1.002 (0.999 - 1.004)	1.015* (1.002 - 1.029)	1 (0.999 - 1.001)	1 (1.000 - 1.000)
10	1.002 (1.000 - 1.005)	1.015* (1.002 - 1.029)	0.999 (0.998 - 1.000)	1 (1.000 - 1.000)
11	1.002 (1.000 - 1.005)	1.013 (0.999 - 1.027)	0.999 (0.998 - 1.000)	1 (1.000 - 1.000)

12	1.002 (1.000 - 1.005)	1.012 (0.998 - 1.026)	0.999 (0.998 - 1.000)	1 (1.000 - 1.000)
13	1.003* (1.000 - 1.006)	1.009 (0.996 - 1.023)	0.999 (0.998 - 1.000)	1 (1.000 - 1.000)
14	1.003* (1.000 - 1.006)	1.004 (0.991 - 1.018)	0.999 (0.998 - 1.000)	1 (1.000 - 1.000)
15	1.003* (1.001 - 1.006)	1.007 (0.994 - 1.021)	0.999* (0.998 - 1.000)	1 (1.000 - 1.000)
16	1.003* (1.000 - 1.006)	1.011 (0.998 - 1.025)	0.999* (0.998 - 1.000)	1 (1.000 - 1.000)
17	1.003 (1.000 - 1.006)	1.011 (0.997 - 1.025)	0.999** (0.998 - 1.000)	1 (1.000 - 1.000)
18	1.002 (1.000 - 1.005)	1 (0.987 - 1.014)	0.999** (0.998 - 1.000)	1 (1.000 - 1.000)
19	1.002 (0.999 - 1.005)	1.004 (0.991 - 1.018)	0.999** (0.998 - 1.000)	1 (1.000 - 1.000)
20	1.002 (0.999 - 1.005)	1.009 (0.995 - 1.022)	0.999** (0.998 - 1.000)	1 (1.000 - 1.000)
21	1.002 (0.999 - 1.005)	1.006 (0.993 - 1.020)	0.998** (0.997 - 0.999)	1 (1.000 - 1.000)
22	1.002 (0.999 - 1.005)	1.002 (0.989 - 1.016)	0.999** (0.997 - 1.000)	1 (1.000 - 1.000)
23	1.002 (0.999 - 1.006)	1.007 (0.994 - 1.020)	0.998** (0.997 - 1.000)	1 (1.000 - 1.000)
24	1.003 (1.000 - 1.006)	1.008 (0.995 - 1.022)	0.998** (0.997 - 0.999)	1 (1.000 - 1.000)
25	1.003 (0.999 - 1.006)	1.011 (0.998 - 1.025)	0.998** (0.997 - 0.999)	1 (1.000 - 1.000)
26	1.003 (1.000 - 1.006)	1.007 (0.993 - 1.021)	0.998** (0.996 - 0.999)	1 (1.000 - 1.000)
27	1.003 (1.000 - 1.006)	1.01 (0.997 - 1.024)	0.998** (0.996 - 0.999)	1 (1.000 - 1.000)
28	1.002 (0.999 - 1.006)	1 (0.987 - 1.014)	0.998** (0.996 - 0.999)	1 (1.000 - 1.000)
29	1.003 (0.999 - 1.006)	1.001 (0.988 - 1.014)	0.997** (0.996 - 0.999)	1 (1.000 - 1.000)
30	1.003 (0.999 - 1.006)	1 (0.987 - 1.013)	0.997** (0.996 - 0.999)	1 (1.000 - 1.000)
**Southeast (SE)**				
0	1.002 (0.999 - 1.005)	1.011 (0.999 - 1.023)	0.999 (0.997 - 1.001)	1 (1.000 - 1.000)
1	1.002 (0.999 - 1.005)	1.004 (0.991 - 1.016)	0.999 (0.997 - 1.000)	1 (1.000 - 1.000)
2	1.002 (0.999 - 1.005)	0.994 (0.982 - 1.006)	0.999 (0.997 - 1.000)	1 (1.000 - 1.000)
3	1.002 (0.999 - 1.006)	0.996 (0.984 - 1.008)	0.999 (0.998 - 1.001)	1 (1.000 - 1.000)
4	1.003 (0.999 - 1.006)	1.005 (0.992 - 1.017)	1 (0.999 - 1.001)	1 (1.000 - 1.000)
5	1.003* (1.000 - 1.007)	1.004 (0.991 - 1.016)	0.999 (0.998 - 1.000)	1.000* (1.000 - 1.000)
6	1.003 (1.000 - 1.007)	1.006 (0.994 - 1.019)	1 (0.999 - 1.000)	1.000* (1.000 - 1.000)
7	1.003* (1.000 - 1.007)	1.006 (0.993 - 1.018)	1 (0.999 - 1.001)	1.000** (1.000 - 1.000)
8	1.003 (1.000 - 1.006)	1.008 (0.996 - 1.021)	1 (1.000 - 1.001)	1.000** (1.000 - 1.000)
9	1.003 (1.000 - 1.006)	1.008 (0.996 - 1.020)	1 (0.999 - 1.000)	1.000** (1.000 - 1.000)
10	1.003* (1.000 - 1.006)	1.01 (0.998 - 1.022)	1 (0.999 - 1.001)	1.000** (1.000 - 1.000)
11	1.003 (1.000 - 1.006)	1.006 (0.994 - 1.018)	1 (1.000 - 1.001)	1.000** (1.000 - 1.000)
12	1.003 (1.000 - 1.006)	0.998 (0.986 - 1.010)	1.001 (1.000 - 1.001)	1.000** (1.000 - 1.000)
13	1.003 (1.000 - 1.006)	1.007 (0.995 - 1.019)	1.001* (1.000 - 1.001)	1.000** (1.000 - 1.000)
14	1.003* (1.000 - 1.006)	1.007 (0.995 - 1.020)	1 (1.000 - 1.001)	1.000** (1.000 - 1.000)
15	1.003* (1.000 - 1.006)	1.012* (1.000 - 1.025)	1 (1.000 - 1.001)	1.000** (1.000 - 1.000)
16	1.003* (1.000 - 1.006)	1.006 (0.994 - 1.018)	1 (0.999 - 1.001)	1.000* (1.000 - 1.000)
17	1.002 (1.000 - 1.005)	1.007 (0.995 - 1.019)	1 (1.000 - 1.001)	1.000* (1.000 - 1.000)
18	1.003 (1.000 - 1.006)	1.003 (0.991 - 1.015)	1 (0.999 - 1.001)	1.000** (1.000 - 1.000)
19	1.003 (1.000 - 1.006)	1.009 (0.997 - 1.021)	1 (0.999 - 1.001)	1.000* (1.000 - 1.000)
20	1.003* (1.000 - 1.006)	1.003 (0.991 - 1.016)	1 (0.999 - 1.001)	1.000** (1.000 - 1.000)
21	1.003* (1.000 - 1.006)	1.006 (0.993 - 1.018)	1 (0.999 - 1.001)	1.000** (1.000 - 1.000)
22	1.003* (1.000 - 1.006)	1.007 (0.995 - 1.020)	1 (1.000 - 1.001)	1.000** (1.000 - 1.000)
23	1.003 (1.000 - 1.006)	1.009 (0.997 - 1.021)	1.001 (1.000 - 1.002)	1.000** (1.000 - 1.000)
24	1.003 (1.000 - 1.006)	0.996 (0.984 - 1.008)	1.001* (1.000 - 1.002)	1.000** (1.000 - 1.000)
25	1.002 (0.999 - 1.005)	0.997 (0.985 - 1.009)	1.001* (1.000 - 1.002)	1.000** (1.000 - 1.000)

26	1.002 (0.999 - 1.005)	0.998 (0.985 - 1.010)	1.001 (1.000 - 1.001)	1.000** (1.000 - 1.000)
27	1.003 (1.000 - 1.006)	1.005 (0.993 - 1.018)	1 (1.000 - 1.001)	1.000** (1.000 - 1.000)
28	1.003* (1.000 - 1.006)	1.009 (0.997 - 1.021)	1 (1.000 - 1.001)	1.000** (1.000 - 1.000)
29	1.003* (1.000 - 1.006)	1.005 (0.992 - 1.017)	1 (0.999 - 1.001)	1.000** (1.000 - 1.000)
30	1.002 (0.999 - 1.005)	1.004 (0.992 - 1.016)	1 (1.000 - 1.001)	1.000** (1.000 - 1.000)
**Subtropical (ST)**				
0	1 (0.995 - 1.006)	1.003 (0.986 - 1.019)	0.999 (0.997 - 1.002)	1 (1.000 - 1.000)
1	1 (0.995 - 1.005)	0.997 (0.981 - 1.014)	1 (0.997 - 1.002)	1 (1.000 - 1.000)
2	1 (0.995 - 1.005)	0.991 (0.975 - 1.008)	1 (0.998 - 1.002)	1 (1.000 - 1.000)
3	1 (0.995 - 1.005)	0.993 (0.977 - 1.009)	1 (0.998 - 1.002)	1 (1.000 - 1.000)
4	1 (0.996 - 1.005)	0.99 (0.974 - 1.007)	1 (0.998 - 1.002)	1 (1.000 - 1.000)
5	1 (0.996 - 1.004)	1.006 (0.990 - 1.023)	1 (0.998 - 1.002)	1 (1.000 - 1.000)
6	0.999 (0.995 - 1.004)	1.008 (0.991 - 1.025)	1 (0.998 - 1.002)	1 (1.000 - 1.000)
7	1 (0.995 - 1.004)	1.006 (0.989 - 1.023)	1.001 (0.998 - 1.003)	1 (1.000 - 1.000)
8	1 (0.996 - 1.004)	1 (0.983 - 1.017)	1.001 (0.998 - 1.003)	1 (1.000 - 1.000)
9	1 (0.996 - 1.004)	0.999 (0.983 - 1.016)	1 (0.998 - 1.003)	1 (1.000 - 1.000)
10	1.001 (0.997 - 1.005)	1.003 (0.987 - 1.020)	1 (0.998 - 1.002)	1 (1.000 - 1.000)
11	1.001 (0.997 - 1.005)	1.004 (0.988 - 1.021)	1 (0.998 - 1.002)	1 (1.000 - 1.000)
12	1.001 (0.997 - 1.005)	1.005 (0.989 - 1.022)	1.001 (0.999 - 1.003)	1 (1.000 - 1.000)
13	1 (0.997 - 1.004)	1.018* (1.001 - 1.035)	1.001 (0.999 - 1.003)	1 (1.000 - 1.000)
14	1.001 (0.997 - 1.004)	1.007 (0.990 - 1.023)	1.001 (0.999 - 1.003)	1 (1.000 - 1.000)
15	1.001 (0.997 - 1.004)	1.002 (0.985 - 1.019)	1.001 (0.999 - 1.003)	1 (1.000 - 1.000)
16	1 (0.997 - 1.003)	1.004 (0.987 - 1.021)	1.001 (0.999 - 1.004)	1 (1.000 - 1.000)
17	1 (0.997 - 1.003)	1.001 (0.985 - 1.018)	1.001 (0.999 - 1.004)	1 (1.000 - 1.000)
18	1 (0.997 - 1.002)	1.007 (0.990 - 1.023)	1.001 (0.999 - 1.003)	1 (1.000 - 1.000)
19	1 (0.997 - 1.003)	1.012 (0.995 - 1.028)	1.001 (0.998 - 1.003)	1 (1.000 - 1.000)
20	1 (0.998 - 1.003)	1.013 (0.997 - 1.031)	1 (0.999 - 1.002)	1 (1.000 - 1.000)
21	1 (0.998 - 1.003)	1.005 (0.988 - 1.022)	1.001 (0.999 - 1.003)	1 (1.000 - 1.000)
22	1 (0.998 - 1.003)	0.995 (0.978 - 1.012)	1.001 (0.999 - 1.003)	1 (1.000 - 1.000)
23	1 (0.998 - 1.003)	0.994 (0.978 - 1.011)	1 (0.998 - 1.002)	1 (1.000 - 1.000)
24	1 (0.998 - 1.002)	1 (0.983 - 1.016)	1.001 (0.999 - 1.003)	1 (1.000 - 1.000)
25	1 (0.998 - 1.002)	1.007 (0.991 - 1.024)	1.002 (1.000 - 1.004)	1 (1.000 - 1.000)
26	1 (0.998 - 1.002)	1.008 (0.992 - 1.025)	1.002 (1.000 - 1.004)	1 (1.000 - 1.000)
27	1 (0.998 - 1.003)	1.013 (0.996 - 1.030)	1.001 (0.999 - 1.003)	1 (1.000 - 1.000)
28	1 (0.998 - 1.003)	1.012 (0.995 - 1.029)	1.001 (0.999 - 1.003)	1 (1.000 - 1.000)
29	1 (0.998 - 1.003)	1.005 (0.988 - 1.023)	1 (0.998 - 1.002)	1 (1.000 - 1.000)
30	1.001 (0.998 - 1.003)	0.994 (0.977 - 1.011)	1.001 (0.998 - 1.003)	1 (1.000 - 1.000)
**Upper Midwest**				
0	1.003** (1.001 - 1.006)	1.027** (1.015 - 1.039)	0.998** (0.996 - 0.999)	1.000** (1.000 - 1.000)
1	1.003** (1.001 - 1.005)	1.021** (1.009 - 1.033)	0.998** (0.996 - 0.999)	1.000** (1.000 - 1.000)
2	1.003* (1.000 - 1.005)	1.014* (1.002 - 1.025)	0.998** (0.996 - 0.999)	1.000* (1.000 - 1.000)
3	1.003* (1.000 - 1.005)	1.014** (1.003 - 1.026)	0.998* (0.997 - 1.000)	1.000* (1.000 - 1.000)
4	1.003* (1.000 - 1.005)	1.017** (1.005 - 1.029)	0.998** (0.996 - 1.000)	1.000* (1.000 - 1.000)
5	1.003* (1.000 - 1.005)	1.023** (1.011 - 1.035)	0.999 (0.997 - 1.000)	1.000* (1.000 - 1.000)
6	1.003* (1.000 - 1.005)	1.026** (1.014 - 1.037)	0.999 (0.997 - 1.000)	1.000* (1.000 - 1.000)
7	1.003* (1.000 - 1.006)	1.030** (1.018 - 1.042)	0.998* (0.997 - 1.000)	1.000* (1.000 - 1.000)

8	1.003* (1.000 - 1.006)	1.022** (1.011 - 1.034)	0.998* (0.997 - 1.000)	1.000* (1.000 - 1.000)
9	1.003* (1.000 - 1.006)	1.014** (1.003 - 1.026)	0.998 (0.997 - 1.000)	1.000* (1.000 - 1.000)
10	1.003* (1.000 - 1.006)	1.012* (1.001 - 1.024)	0.999 (0.997 - 1.001)	1.000* (1.000 - 1.000)
11	1.003** (1.001 - 1.006)	1.013* (1.002 - 1.025)	0.998* (0.997 - 1.000)	1.000* (1.000 - 1.000)
12	1.003** (1.001 - 1.006)	1.013* (1.001 - 1.024)	0.998* (0.997 - 1.000)	1.000* (1.000 - 1.000)
13	1.003** (1.001 - 1.006)	1.017** (1.006 - 1.029)	0.999 (0.997 - 1.000)	1.000** (1.000 - 1.000)
14	1.003** (1.001 - 1.006)	1.019** (1.007 - 1.031)	0.999 (0.997 - 1.000)	1.000** (1.000 - 1.000)
15	1.004** (1.001 - 1.006)	1.014** (1.003 - 1.026)	0.999 (0.997 - 1.000)	1.000** (1.000 - 1.000)
16	1.004** (1.001 - 1.006)	1.013* (1.001 - 1.024)	0.999 (0.997 - 1.000)	1.000** (1.000 - 1.000)
17	1.004** (1.001 - 1.006)	1.011 (1.000 - 1.023)	0.999 (0.998 - 1.000)	1.000** (1.000 - 1.000)
18	1.004** (1.001 - 1.006)	1.013* (1.001 - 1.025)	0.999 (0.997 - 1.000)	1.000** (1.000 - 1.000)
19	1.004** (1.002 - 1.007)	1.016** (1.005 - 1.028)	0.999 (0.998 - 1.000)	1.000** (1.000 - 1.000)
20	1.004** (1.001 - 1.006)	1.021** (1.009 - 1.033)	0.998* (0.997 - 1.000)	1.000** (1.000 - 1.000)
21	1.004** (1.002 - 1.007)	1.025** (1.013 - 1.037)	0.999 (0.997 - 1.000)	1.000** (1.000 - 1.000)
22	1.005** (1.002 - 1.007)	1.022** (1.011 - 1.034)	0.999 (0.997 - 1.000)	1.000** (1.000 - 1.000)
23	1.004** (1.002 - 1.007)	1.020** (1.008 - 1.031)	0.999 (0.998 - 1.001)	1.000** (1.000 - 1.000)
24	1.004** (1.002 - 1.007)	1.018** (1.007 - 1.030)	0.999 (0.998 - 1.001)	1.000** (1.000 - 1.000)
25	1.004** (1.001 - 1.007)	1.013* (1.002 - 1.025)	0.999 (0.998 - 1.000)	1.000** (1.000 - 1.000)
26	1.004** (1.001 - 1.006)	1.019** (1.008 - 1.031)	0.999* (0.997 - 1.000)	1.000** (1.000 - 1.000)
27	1.004** (1.001 - 1.006)	1.021** (1.010 - 1.033)	0.998* (0.997 - 1.000)	1.000** (1.000 - 1.000)
28	1.004** (1.001 - 1.006)	1.019** (1.008 - 1.031)	0.998* (0.997 - 1.000)	1.000** (1.000 - 1.000)
29	1.004** (1.001 - 1.006)	1.01 (0.999 - 1.022)	0.998* (0.997 - 1.000)	1.000** (1.000 - 1.000)
30	1.004** (1.001 - 1.006)	1.01 (0.999 - 1.022)	0.999* (0.997 - 1.000)	1.000** (1.000 - 1.000)
